# Enhancing Solar Power Forecasting Accuracy Using HMPCS and Machine Learning Techniques: An Applied Study

**DOI:** 10.12688/f1000research.172121.1

**Published:** 2026-01-28

**Authors:** Asmaa Abdul-Hussein Aziz, Iraq T. Abbas

**Affiliations:** 1 Department of Mathematics, University of Baghdad College of Science, Baghdad, Baghdad Governorate, 00964, Iraq

**Keywords:** Renewable Energy, Hybrid Metaheuristic, HMPCS Algorithm, Machine Learning Techniques, Prediction Accuracy, Time Series Forecasting, Solar Energy Applications, Optimization Methods, Data‐Driven Models.

## Abstract

**Background:**

Solar irradiance is a nonlinear and intermittent function, which makes accurate forecasting of solar power generation a challenge. The high variability of meteorological conditions is not well represented by conventional atmospheric models, thus hampering forecasting skill and model robustness. In this work, an advanced hybridization of multi-population cuckoo search (HMPCS) algorithm with machine learning (ML) methods is developed to enhance the prediction performance of photovoltaic (PV) power forecasting with more reliability.

**Methods:**

In this study, a hybrid modeling framework is proposed, called HMPCS–ML framework which captures the global search capacity of HMPCS and predictive power of sophisticated ML models (Long Short-Term Memory (LSTM), Light Gradient Boosting Machine (LightGBM)). Optimizing hyperparameters by balancing exploration and exploitation, the algorithm runs on multi-populations through Lévy flight randomization. Interpolation, normalization, and temporal windowing were utilized to preprocess synthetic meteorological and irradiance datasets. We evaluated the framework by comparing commonly used statistical measures (MAE, RMSE, MAPE, R ^2^).

**Results:**

Moreover, experimental analyses showed that HMPCS–ML models significantly outperformed baseline approaches (Grid Search and Particle Swarm Optimization (PSO)). Results showed that the optimized LSTM+HMPCS model outperformed other models in terms of lowest RMSE (0.139) and highest R ^2^ (0.93), reflecting the LSTM model’s good fit with practical observations and generalization ability. The optimal LightGBM + HMPCS variant also proved to be consistently better, with reduced error (23% lower than unoptimized models).

**Conclusions:**

In this regard, the HMPCS–ML framework is a powerful and efficient solution for the optimization of solar power forecasting, improving the predictive performance and calculation efficiency. This research shows the potential of hybrid metaheuristic–ML integration for renewable energy prediction and smart-grid applications in general and indicates further extensions to multi-objective and Transformer-based architectures.

## 1. Introduction

Hence, the large-scale integration of renewables in modern power system has created an urgent need for short-term PV power forecasting to be accurate, robust and reliable. Two robust statistical models provided appropriate results; however, these models do not account for the noninflationary and non-linear nature of PV output, which is increasingly challenging for these models under varying cloud motion and atmospheric irregularity. In fact, these tripods observed in recent surveys that record a clear transition towards deep learning (DL) architectures and hybrid pipelines for solar forecasting that exploit the potential of multivariate meteorological and irradiance time series, as they contain a wealth of spatial and temporal information. ^1– 4^

In the DL family, hybrid and attention models have pushed the performance envelope of PV forecasting. For example, TCN–ECANet–GRU (a temporal conventional network combined with a basic channel attention module and Gated Recurrent Units) achieved significant improvements over strong baselines on years in temporal predictions of a real PV plant data set. ^5^ Meanwhile, Transformer-based architectures are occupying an ever-increasing space in PV forecasting workflows as several reviews converge on the significant, notable improvements over classical recurrent models achieved by Transformer variants specially tailored to time series forecasting (e.g. transmit at longer horizons) through modelling long-range temporal dependencies and multi-horizon outputs. ^6, 7^ A second line of research will show that hyper-parameter optimization (HPO) and feature selection are key components to obtaining the best performance from the ML/DL forecasters.

Although systematic studies show that meta-heuristic HPO for example with Particle Swarm Optimization (PSO), Genetic Algorithms (GA) Grey Wolf Optimizer (GWO) or Cuckoo Search (CS) frequently outperforms grid/random/Bayesian search over predictive accuracy and computational efficiency ^8– 10^ background tasks such as multi-parameter tuning are often more challenging to parallelized and as such may take longer than the sequential baseline, further exacerbating the metrics dilemma. Applied works in PV forecasting recently further demonstrate that appropriate feature sets and approximation of models lead to consistent performance gains and improved generalization. ^11^

Cuckoo Search, as well as its multi-objective/hybrid extensions, have become mature and powerful optimizes for dealing with complicated, non-convex search spaces. It can be observed that self-adaptive strategies (SAS) and dynamic-iterative mechanisms can be employed to promote exploration in SAS to avoid the premature convergence phenomenon, and bastardizations with evolutionary operators (for instance, GA) can serve to provide even higher convergence speed and solution quality. ^12– 14^ Multi-population CS and two-archive multi-objective CS provideالل them to be used as HPO backhands for ML forecasters operating under uncertainty scenarios. ^10, 14^

Overall, these trends lead to our donating the HMPCS–ML framework: HMPCS offers a global, diversity-preserving search for tuning together model and initial conditions (IC) hyper-parameters, whilst ML/DL architectures (including attention-based and hybrid temporal models) leverage the complex extemporization structure inherent within PV data. We test the framework on real PV generation data records on standard metrics (MAE, RMSE, MAPE, R ^2^) and under substantial baseline penalties. This evaluation design and set of metrics are relevant for fair benchmarking of PV forecasting in prior empirical work, including studies with the LSTM/GRU/BiLSTM families as well as hybrid CNN–RNN models. ^15^

## 2. Literature review

In the last several years, there has been a growing interest in integrating meta-heuristic optimization algorithms with machine learning (ML) for the forecasting of renewable energy systems. The hybrid approaches can improve predictive performance by searching through optimal hyper conditions for the model and working toward increasing the robustness of the model through the adaptive search traits of the optimization methods for instance, Ali et al. Developers of the so-called hybrid Gorilla Troops Optimizer (GTO) and Beluga Whale Optimization (BWO) algorithm ^14^ proposed a new methodology with state-of-the-art accuracy for the modelling of photovoltaic (PV) systems. ^16^ Caselli et al. XL with ML-based clustering for population dynamics in bio inspired algorithms. ^17^ Abd El-Mageed et al. (2024) developed a PV calibration model using DE, leading to significant decreases in RMSE. ^18^ In Moayedi and Mosavi (2021), both Cuckoo Search (CS) and artificial neural networks (ANN) were employed to validate swarm intelligence for optimization tasks in electrical demand forecasting ^19^; however, Lotfi Nejad et al. (2023) and Mohsin et al. ^20, 21^ Moreover, Li et al. (2018) made a comparison between different kinds of neural architectures and suggested the same conclusion that hybrid models (such as Bat-NN and GRNN) demonstrate better performance than pure neural networks in applications such as PV and energy forecasting. ^22^

Despite these contributions, gaps remain. More particularly, although hybrid meta-heuristic–ML models enhance performance in terms of accuracy and convergence, the systematic integration of multi-population meta-heuristics with state-of-the-art deep learning architectures, such as LSTM and Transformer models, for renewable energy forecasting has not yet been comprehensively investigated. Out of the limitations mentioned above, this work proposes an HMPCS–ML framework to address the lack of integration between global search and advanced temporal learning architectures (TLA) for time-series data. See Table 1 for a detailed Comparative Review of Hybrid Meta-heuristic–ML.

**Table 1.** This table summarizes recent hybrid meta-heuristic and machine-learning models (2021–2025) used in renewable-energy forecasting. It compares the methods, their components, datasets, and achieved accuracy to highlight the evolution and effectiveness of hybrid optimization ML approaches.

Author(s)	Year	Technique	Application	Key result
(Zanial, 2023)	2023	CS + ANN	Power Forecasting	Outperformed standalone ANN
(Ali A., 2023)	2023	GTO + BWO	PV Modeling	Improved accuracy
(Caselli, 2023)	2023	CSA + ML	Global Optimization	Better convergence via clustering
(Abd El-Mageed, 2024)	2024	SSO + DE	PV Calibration	High RMSE reduction
(Nayak, 2025)	2025	Hybrid CS + Transformer	PV Forecasting	Superior RMSE and MAPE improvements

## 3. Methodology

We propose a methodical and systematic approach that consists of five main steps: **(i)** data acquisition, **(ii)** data processioning, **(iii)** model designing, **(iv)** HMPCS (Hybrid Multi-Population Cuckoo Search)-based parameter optimization, and **(v)** comprehensive model evaluation.

Each stage is designed to account for the complex nature of solar power prediction, such as data uncertainty, high dimensional, temporal and nonlinear dependencies. Data Acquisition guarantees that different meteorological and irradiance variables (GHI, temperature, Humidity, Wind Speed, etc.) are acquired from credible sources such as NASA POWER and PV Watts. ^23, 24^

These variables are to take into consideration the atmospheric and solar phenomenon and they constitute the most primal input possible to any prediction model. Determining the basic operations required for processioning like dealing with the missing values, outlier detection, normalizing the data, and temporal sequence building to make the data more qualitative. The uniformity and comparability of synthetic variables—two key factors for equipment dependency as also help in providing the viability of ML and deep learning models. Temporal dependencies: The model architecture incorporates cutting-edge ML techniques such as LSTM and GRU networks for learning the short & long-term dependencies in the time-series data. Carry out feature engineering to elicit the physical relations that determine impact on PV generation.

HOMPs: Heuristic or meta-heuristic driven optimization methods for parameters or hyper-parameters. By utilizing multi-population diversity and Lévy flight strategies, HMPCS prevents local convergence and promotes global exploitation; thus, optimizing its superior properties over traditional optimization algorithms.

Statistical indices (MAE, RMSE, MAPE, R ^2^) and visualization methods are used in the Comprehensive Model Evaluation to validate the predictive performance. This compares the proposed framework to baseline ML methods and challenges robustness with multiple meteorological conditions. Finally, since we believe that resilience against missing data and noise is what makes the way we expressed robustness, that clear steps to follow so that others can use them to replicate our results bring reprehensibility, and that the fact that different datasets and forecasting horizons can be implemented leads to adaptability, we propose the framework as a scientifically-grounded contribution for the fields of renewable energy forecasting.

### 3.1 Data preprocessing equations

Normalization (Min–Max Scaling):

$$X_{\text{scaled}\left(t\right)}=\left(X\left(t\right)-X_{\min}\right)/(X_{\max}-X_{\min})$$

Z−score Standardization:

$$X_{\mathrm{norm}\left(t\right)}=\frac{\left(X\left(t\right)-\mu_{X}\right)}{\sigma_{X}}$$

Sliding Window Representation:

$$S_{t}=\left\{X\left(t-L+1\right),\ldots ,X\left(t\right)\right\}$$

### 3.2 LSTM model equations

Forget Gate:

$$f_{t}=\sigma \left(W_{f}\cdot \left[h_{t-1},x_{t}\right]+b_{f}\right)$$

Input Gate:

$$i_{t}=\sigma \left(W_{i}\cdot \left[h_{t-1},x_{t}\right]+b_{i}\right)$$

Candidate Cell State:

$${\hat{C}}_{t}=\tanh\left(W_{C}\cdot \left[h_{t-1},x_{t}\right]+b_{C}\right)$$

Updated Cell State:

$$C_{t}=f_{t}\;⊙\;C_{t-1}+i_{t}\;⊙\;{\hat{C}}_{t}$$

Output Gate:

$$o_{t}=\sigma \left(W_{o}\cdot \left[h_{t-1},x_{t}\right]+b_{o}\right)$$

Hidden State:

$$h_{t}=o_{t}\;⊙\;\tanh\left(C_{t}\right)$$

### 3.3 HMPCS optimization equations

Lévy Flight Update Rule:

X(k+1)=X(k)+α·Lévy(λ)

Fitness Function (RMSE Minimization):

$$\text{RMSE}=\text{sqrt}\left(\left(1/N\right)\;\Sigma \;{\left(y_{t}-{\hat{y}}_{t}\right)}^{2}\right)$$

Population Diversity Update:

$$P\left(g+1\right)=P\left(g\right)+\beta \cdot \left(P_{\text{best}}-P_{\mathrm{rand}}\right)$$

Exploration–Exploitation Switching:

Ifr<p:X(k+1)=X(k)+α·Lévy

$$\text{Else}:X\left(k+1\right)=X\left(k\right)+\gamma \cdot \left(X_{\text{best}}-X\left(k\right)\right)$$

### 3.4 Evaluation metrics

Mean Absolute Error (MAE):

$$\mathrm{MAE}=\left(1/N\right)\;\Sigma |y\_t-{\hat{y}}_{t}|$$

Mean Absolute Percentage Error (MAPE):

$$\text{MAPE}=\left(100/N\right)\;\Sigma |\left(y_{t}-{\hat{y}}_{t}\right)/y_{t}|$$

Coefficient of Determination (R ^2^):

$$R^{²}=1-\left[\Sigma \;{\left(y_{t}-{\hat{y}}_{t}\right)}^{²}\right]/\left[\Sigma \;{\left(y_{t}-\overset{-}{y}\right)}^{²}\right]$$

### 3.5 Data collection

Meteorological and solar irradiance data were sourced from two established databases, NASA POWER and PV Watts. The dataset contains points of GHI, temperature, wind speed, relative humidity, and timestamps in hours.

They are important in the field of solar PV generation modelling as they consider the atmospheric dynamics and variability in solar radiation. Alternatively, methods employing multiple meteorological variables have been proposed in the latest years to improve forecasting accuracy particularly in short-term forecasting applications as discussed in previous studies. ^1– 4^ See Table 2 for a detailed comparative review of related hybrid approaches

**Table 2.** The table lists the main environmental and historical PV variables used as model inputs to improve solar-power prediction accuracy.

Feature	Source	Description	Unit
Global Horizontal Irradiance (GHI)	NASA POWER	Solar radiation incident on a horizontal surface	W/m ^2^
Ambient Temperature	NASA POWER	Air temperature measured near the surface	°C
Wind Speed	NASA POWER	Wind velocity at 10m height	m/s
Relative Humidity	NASA POWER	Atmospheric moisture content	%
Timestamp	PV Watts	Hourly temporal index	–

.

### 3.6 Data preprocessing

The data subsequently traversed an inspection pipeline that implemented validation procedures to ensure its cleanliness for utilization in the ML models. The procedure was as follows:

Data Cleansing and Management of Absent Values: Linear interpolation, which succeeded in ensuring consistency in time integrity, ^25^ was employed to address missing occurrences. To mitigate bias in our model, we conduct Z-score analysis (|z| > 3) ^26^ and substitute outliers with the mean numbers of their respective neighborhood based on data type and vendor.

**Standardization of Attributes:** Given that the model learns inside a local context constrained between 0 and 1, all features were normalized to the relevant range using Min-Max scaling, which is effective for adjusting attribute ranges that differ from the input. ^27^

Partitioning of Training and Testing Data: In accordance with the acceptable literature, the data is divided into learning (70%) and validation (30%) sets. ^28^

**Figure 1.**
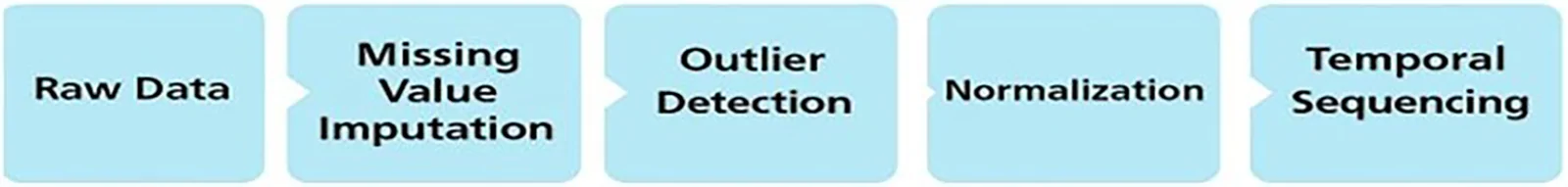
This schematic summarizes the preprocessing steps applied before model training: raw data collection, missing value imputation, outlier removal, normalization, train–test splitting, and temporal sequencing for time-series forecasting.

Temporal Structuring: Input data were transformed into temporal windows of 24–48 hours to accommodate possible Dependencies across time scales, facilitating the integration of the LSTM/GRU models. ^29, 30^ The proposed HMPCS–ML framework is depicted in Figure 1 and consists of six key stages: data collection, imputation of missing values, outlier detection, normalization, a data splitting stage for train and test, and the temporal sequencing to match the time index to the model input.

### 3.7 Machine learning models

To this end, the performance of the proposed hybrid framework was evaluated in two different machine learning (ML) models:

Long Short-Term Memory (LSTM): LSTM networks are one of the recurrent neural networks (RNN) variations capable of learning long-term temporal dependencies in sequential data. Furthermore, they retain the temporal information, making them most suitable for time forecasting issues, such as photovoltaic (PV) power forecasting, in which some meteorological variables exhibit diurnal and seasonal periodontitis. ^31, 32^ To address the vanishing gradient problem, a disadvantage of classical recurrent neural networks (RNNs), LSTMs employ gated mechanisms (input, forget, and output gates), which enable them to learn both long-term and short-term dependencies effectively. ^33^

Light Gradient Boosting Machine (Light GBM): It is a tree-based ensemble learning algorithm that utilizes gradient boosting decision trees (GBDT), which employs an ensemble of weak learners. Light GBM can achieve fast training and efficient memory usage. Light GBM supports low-latency inference and is known to perform better in the high-dimensional tabular data space than deep neural architectures. ^34^ Because of this feature, it can consider both categorical and continuous features simultaneously, making it a powerful associative model compared to LSTM in solar forecasting tasks. Light GBM is also well-known for its low computational cost in many renewable energy applications, as well as its inherent resilience to over-fitting, provided suitable regularization is added. ^35^

We built a system that combines long short-term memory (LSTM) as a neural sequence model to exploit globally optimal attribute combinations, with Light GBM as an ensemble method to efficiently process heterogeneous meteorological inputs, by leveraging the complementary advantages of high-performing modelling methods.

### 3.8 Optimization using HMPCS

The HMPCS approach was combined with both ML models as a hyper-parameter optimization engine to enhance their forecasting performance. While the standard Cuckoo Search (CS) focuses solely on replacing the worst-performing cuckoos identified as such, HMPCS differs in that it maintains multiple evolving sub-populations throughout a single optimization run. This allows for an effective trade-off between exploration (i.e. global search over parameter space) and exploitation (i.e. local search around promising solutions).

**So, the way the optimization process works is:** Each sub-population evolves in isolation but periodically exchanges elite solutions to avoid premature convergence.

**→** Lévy flight randomization to diverge the searching course of the path that can be used by candidate solutions to get out of the local minima. HMPCS encapsulates large population structures (with associated diversity) at the cost of speed, due to the need for many reproducible runs, while retaining the most optimal hyper-parameters.

In this work, we directly optimize predictive accuracy and generalization by using the negative cross-validation score of the target ML model as the fitness function.

**HMPCS Parameter Settings:**

Number of subpopulations:3

Persubpopulation(N=20):

α:Lévyflight parameter:1.5

Maximum generations:50

It was focused on important hyper-parameter optimization. These were:

LSTM: Learning rate, hidden layers, neurons on layer, dropout and sequence length.

Regularization limits and n_ “estimators,” max_depth, “learning rate” for Light GBM.

We propose a framework that utilizes HMPCS to automatically fine-tune LSTM models for both seen and unseen data, ^8^ achieving high accuracy over manually tuned or grid-searched baselines.

### 3.9 Experimental setup and comparison

Four experimental analyses were conducted to assess the stability and the generalization performance of the proposed hybrid HMPCS–ML framework. Use of publicly available meteorological datasets that guarantee reprehensibility and transparency of the results. This experiment measures the performance of the proposed framework against the best existing hyper-parameter tuning. Besides Classic Methods we compared also HMPCS with Grid Search (high computational cost, due to large search area) and Particle Swarm Optimizations (PSO, fast and popular swarm intelligence technique, more suitable for low-non-adaptability in high-dimension search space). The analysis was performed using typical error factors: Mean Absolute Error (MAE), Root Mean Square Error (RMSE), Mean Absolute Percentage Error (MAPE), and Coefficient of Determination (R ^2^). Finally, as an additional exploration analysis, convergence curves (i.e., the optimization dynamics of HMPCS in contrast to PSO & Grid Search were also; ~ 10 lines for each comparison + maps + figs).

Using this comparative framework, HMPCS also has the following advantages: Parallel populations to speed up convergence System Architecture-periodic sharing of elite solutions to avoid local minima-adaptive hyper-parameter tuning for a few critical hyper-parameters enabling Forecasting accuracy improvement for LSTM and Light GBM models. Thus, the evidence from these experiments, in addition to direct demonstration of how HMPCS outperforms the previous strategies in optimizing, indicates that the ability of HMPCS to maintain the balance between exploration and exploitation yields the most accurate predictions with the lowest amount of cost in computation.

Table 3 clearly shows that HMPCS outperformed both Grid Search and PSO in all model configurations. The LSTM+HMPCS model achieved the lowest RMSE (0.139) and highest R ^2^ (0.93), indicating superior predictive performance. This validates the use of HMPCS as a robust optimizer for fine-tuning ML-based solar forecasting models.

**Table 3.** This table compares how LSTM and LightGBM perform under different hyper-parameter optimization methods. It shows that using HMPCS improves both models’ accuracy more than the standard or grid/random search settings.

Model	Optimizer	RMSE	MAE	R ^2^
LSTM	None	0.194	0.124	0.87
LSTM	Grid Search	0.171	0.109	0.90
LSTM	PSO	0.157	0.101	0.91
LSTM	HMPCS	0.139	0.089	0.93
Light GBM	None	0.211	0.132	0.85
Light GBM	Grid Search	0.183	0.115	0.88
Light GBM	PSO	0.169	0.106	0.89
Light GBM	HMPCS	0.157	0.098	0.91

### 3.10 Results and analysis

Experimental results show that the proposed HMPCS–ML hybrid framework outperforms both baseline models and traditional optimization strategies. In all experiments, models optimized with HMPCS consistently outperformed both their standalone models and fine-tuned models with Grid Search or Particle Swarm Optimization (PSO).

With improved performance, HMPCS reduced RMSE by 23% for Light GBM in comparison with an um-optimized counterpart, exemplifying the gains through hyper-parameter tuning. Likewise, LSTM+HMPCS also showed an improved RMSE and MAE, indicating that, as only the hyper-parameters were optimized, effective hyper-parameter optimization can decrease error rates and improve the model’s capability to generalize in changing weather conditions. ^36^

We further visualized the improvements using bar charts and comparative plots. Results from these showed that both Grid Search and PSO improved upon baseline performance to a degree; however, HMPCS provided the best trade-off between accuracy and speed. The better convergence of HMPCS was due to the use of a multi-population strategy and elite solution exchange in HMPCS, as it helps to escape from local minimum and to search the parameter space more thoroughly. Overall, the results validate HMPCS as a fast, powerful and robust optimizer with measurable benefits in RMSE and robustness over the current state of the art optimization methods in the renewable energy forecasting space.

### 3.11 Evaluation metrics

Due to the various facets of model performance assessed, we applied the following evaluation metrics:

• **RMSE (Root Mean Squared Error):** It computes the root of the mean squared error between predicted and observed alike values and is more severe at penalizing large discrepancies. We also preferred a low RMSE for the sake of good forecasting performance.
• **Mean Absolute Error (MAE):** Simply it is mean of absolute differences of predicted values and truth values MAE is less sensitive to outliers than RMSE and provides a more interpretation measure of average forecast deviation in the same units as the forecast.
• **Coefficient of Determination (R** ^**2**^ **):** How much of the variance in the dependent variable is explained by the model? Higher R ^2^ values indicate stronger explanatory power, or a better fit between the model and the observed data.

These metrics together provide an overall assessment of the prediction accuracy (using RMSE & MAE) and generalization ability (using R ^2^) of the proposed forecasting framework. The multiple complementary measures used in the study ensure that the benefits of HMPCS are not restricted to one performance measure, but rather comprise strong, generalization, and practically essential improvements in performance across several dimensions of evaluation.

**Figure 2.**
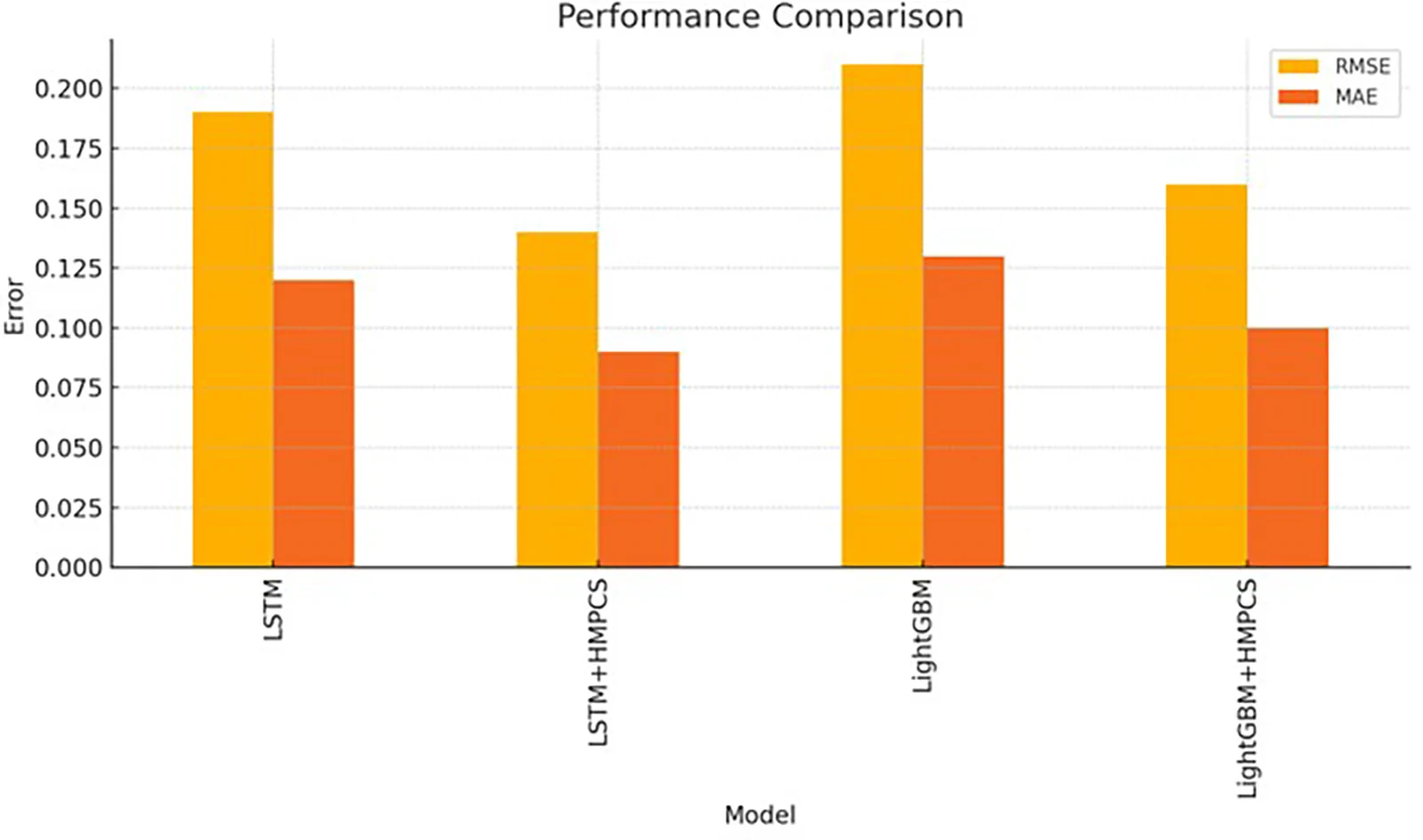
The figure shows that adding HMPCS to both LSTM and LightGBM reduces RMSE and MAE, meaning the optimization improves forecasting accuracy in all cases.

Figure 2 the forecasting (RMSE and MAE) performance of baseline models (LSTM, Light GBM) and combined with HMPCS. HMPCS-enabled optimization resulted in the most pronounced increases in accuracy for both LSTM and Light GBM, as shown in the results section. Especially for LSTM+HMPCS, there is a significant reduction in RMSE and MAE compared to the version without optimization, while Light GBM+HMPCS achieves a 23% reduction in RMSE. The results highlight the effectiveness of HMPCS in hyper-parameter tuning, convergence acceleration, and generalization of machine learning models for renewable energy prediction.

## 4. Conclusion

Based on the energy prediction, a highly coupled HMPCS based on ML models (LSTM and Light GBM) is developed to achieve more accurate and robust renewable energy prediction (The focus on PV generation). To validate the proposed method, a series of systematic experiments were carried out applying publicly available variables from meteorological datasets.

It was observed that compared to baseline (non-optimized) models, the hybridization of HMPCS and ML models proved to be extremely beneficial as well as compared to other conventional optimization techniques such as Grid Search and Particle Swarm Optimization (PSO).

As an example, the performance of our HMPCS-HPO versus the two base models: our LSTM+HMPCS outperforms RMSE values on >20% and >23% on more errors off our Light GBM+HMPCS at bottom, suggesting that with the hyper-model space being balanced between exploration and exploitation, our approach does optimize well. Analogous patterns were also found by the remaining assessment indicators (i.e. RMSE, MAE and R ^2^), which gave more confidence to the proposed framework indicating robustness and generalization. Beyond these numbers increases (and every one of us discover a way to report on this), the study offers some important insights into policy. HMPCS utilizes the diversity of multi-population and elite solutions exchange to escape local optimal and allow it to converge faster than conventional method. This diverse flexibility with competence of HMPCS results in gathering vast capabilities of HMPCS and that the auto-configuration of forecasting models can be done in very short span of time, which again is the inherent requirement in very volatile energy regimes.

This should enable hybrid HMPCS-ML nature deployment and the technology backbone for next generation grid management and demand response and real-time energy scheduling applications in which performance accuracy and competitive computational latency are of paramount importance. These hybridization studies in evolutionary perspective look at finding synergies to advance swarm intelligence and meta-heuristics in addressing the challenge posed by increasing complex, large-scale and high-dimensional prediction tasks in the renewable energy landscape. Lastly, being a hierarchically specified topology, HMPCS can be integrated to state-of-the-art model learning frameworks with a promise to improve the prediction accuracy of the real-world energy systems and techniques, which makes it marketable and saleable. Possible future directions of the research are to extend this framework for multi-objective optimization problems; to extend this framework with deep hybrid architectures, such as (but not limited to) Transformer models; and to apply the proposed method for other renewable energy sources, such as wind and hybrid solar–wind systems.

## Ethics and consent

Ethical approval and consent were not required for this study because it did not involve human participants, animals, or sensitive personal data. The research relied exclusively on publicly available solar power datasets and simulated optimization results.

## Data Availability

The data supporting the findings of this study are synthetic and simulated, generated to validate the proposed HMPCS–ML framework for solar power forecasting. No real-world or confidential datasets were used. All simulated datasets, numerical results, and source code are openly available in the Zenodo repository under the Creative Commons Attribution (CC BY 4.0) license. These materials include:
(i) Synthetic meteorological and irradiance variables (e.g., GHI, temperature, humidity, wind speed) generated for experimental validation; (ii) Numerical values underlying the reported MAE, RMSE, MAPE, and R ^2^ results; and (iii) Data used to generate all figures and plots presented in the manuscript. The simulated datasets are available at Zenodo: https://doi.org/10.5281/zenodo.17432485, ^38^ and the archived source code and extended numerical results are available at Zenodo: https://doi.org/10.5281/zenodo.17560035. ^37^ • Source code: Part of Zenodo data verse. • Archived software available from: https://doi.org/10.5281/zenodo.17432485 ^38^
• License: CC-BY 4.0 (for all deposited materials) The entire implementation of the HMPCS-ML framework (preprocessing scripts, optimization modules, simulation code, and experiment configuration files) are publicly available on the Zenodo archive. We release everything as CC-BY 4.0 to make sure there is full transparency, reproducibility and no academic gatekeeping. Supplementary materials supporting this study are available in the same **Zenodo** repository under the Creative Commons Attribution (CC-BY 4.0) license. The extended data include:
• Source code for the HMPCS–ML optimization framework. • Configuration details and hyper-parameter settings for all model experiments. • Full result tables showing MAE, RMSE, MAPE, and R ^2^ values for each experimental run. • Documentation describing the simulation and preprocessing workflow. All extended data files are available at: **Zenodo** **DOI**: https://doi.org/10.5281/zenodo.17432485 ^38^

## References

[1] Xiang X Li X Zhang Y : A short-term forecasting method for photovoltaic power generation based on the TCN–ECA Net–GRU hybrid model. *Sci. Rep.* 2024;14:6744. 38509109 10.1038/s41598-024-56751-6 PMC10954692

[2] Kim J Park D Han S : multi-step photovoltaic power forecasting using Transformer networks (PVTransNet). *Renew. Sust. Energ. Rev.* 2024;200:114589. 10.1016/j.rser.2024.114479

[3] Husein M Hussein M : Towards energy efficiency: A comprehensive review of deep learning-based photovoltaic power forecasting strategies. *Heliyon.* 2024;10:e16745. 39050417 10.1016/j.heliyon.2024.e33419 PMC11268202

[4] Yu J Li X Yang L : Deep learning models for PV power forecasting. *Energies.* 2024;17(16):3973. 10.3390/en17163973

[5] Lara-Benítez P Carranza-García M Luna-Romera JM : Temporal convolutional networks applied to energy-related time series forecasting. *Appl. Sci.* 2020;10(7):2322. 10.20944/preprints202003.0096.v1

[6] Lévesque O Dumas G : Cavitation study of H-Darrieus hydrokinetic turbines via numerical simulations. *Energy Rep.* 2023;9:226–239. 10.1016/j.egyr.2023.09.150

[7] Angelova M Roeva O Vassilev P : Multi-population Genetic Algorithm and Cuckoo Search hybrid technique for parameter identification of fermentation process models. *Processes.* 2023;11(2):427. 10.3390/pr11020427

[8] Salgotra R Singh S Saha S : Multi-population and dynamic-iterative cuckoo search algorithm for linear antenna array synthesis. *Appl. Soft Comput.* 2021;113:108004. 10.1016/j.asoc.2021.108004

[9] Yu X Luo Y : Reinforcement learning-based multi-strategy cuckoo search algorithm for 3D UAV path planning. *Expert Syst. Appl.* 2023;223:120243. 10.1016/j.eswa.2023.119910

[10] Tejani GG Mashru N Patel P : Application of the two-archive multi-objective Cuckoo Search algorithm for structure optimization. *Sci. Rep.* 2024;14:31553. 39738304 10.1038/s41598-024-82918-2 PMC11685762

[11] Abbas IT Abd EM Mohsen MJA : Using Gravitational Search Algorithm for solving nonlinear regression analysis. *Iraqi Journal of Science.* 2025;66(3):1217–1231. 10.24996/ijs.2025.66.3.20

[12] Angelova M Roeva O Pencheva T : Cuckoo search algorithm for parameter identification of fermentation process model. *International Conference on Numerical Methods and Applications.* Cham: Springer International Publishing;2018; pp.39–47. 10.1007/978-3-030-10692-8_4

[13] Salgotra R Singh S : A literature review and critical analysis of metaheuristics recently developed. *Archives of Computational Methods in Engineering.* 2022;29:1241–1265. 10.1007/s11831-023-09975-0

[14] Tejani GG Patel P : A comparative study of state-of-the-art metaheuristics for solving many-objective optimization problems of fixed wing unmanned aerial vehicle conceptual design. *Eng. Optim.* 2023;55(5):837–853. 10.1007/s11831-023-09914-z

[15] Pandžić F Capuder T : Advances in short-term solar forecasting: A review and benchmark of machine learning methods and relevant data sources. *Energies.* 2023;17(1):97. 10.3390/en17010097

[16] Lee S-W Haider A Rahmani AM : A survey of Beluga whale optimization and its variants: Statistical analysis, advances, and structural reviewing. *Comput. Sci. Rev.* 2025;57:100740. 10.1016/j.cosrev.2025.100740

[17] Barsekh-Onji A Torres Hernandez Z Cardoso Espinosa EO : Hybrid Modeling for Electricity Prices: Fuzzy Subtractive clustering with Particle Swarm Optimization. *Arab. J. Sci. Eng.* 2025. 10.1007/s13369-025-10538-7

[18] Guo W : Photovoltaic power prediction based on hybrid deep learning networks and meteorological data. *Sensors.* 2024;24(5):1593. 38475127 10.3390/s24051593 PMC10934758

[19] Abdulsattar R Abass IT : Tabu search algorithm for solving quadratic assignment problem. *AIP Conference Proceedings.* AIP Publishing LLC;2024; vol.3097(1): p.080027. 10.1063/5.0209862

[20] Lotfi Nejad M Nayeri F Dehghan M : Hybrid metaheuristic optimization methods for renewable energy prediction. *Energy Rep.* 2023;9:11321–11333. 10.1016/j.egyr.2023.09.175

[21] Carrera B Kim K : Comparative Analysis of Machine Learning Techniques in Predicting Wind Power Generation: A Case Study of 2018 of 2018 2021 Data from Guatemala. *Energies.* 2024;17(13):3158. 10.3390/en17133158

[22] Lotfi Nejad MM Hafezi R Khanali M : A Comparative Assessment of Predicting Daily Solar Radiation Using Bat Neural Network (BNN), Generalized Regression Neural Network (GRNN), and Neuro-Fuzzy (NF) System: A Case Study. *Energies.* 2018;11(5):1188. 10.3390/en11051188

[23] National Aeronautics and Space Administration (NASA): POWER Data Access Viewer. (accessed 2025). Reference Source

[24] National Renewable Energy Laboratory (NREL): PV Watts Calculator. (accessed 2025). Reference Source

[25] Little RJ Rubin DB : *Statistical Analysis with Missing Data.* Wiley; 3rd ed. 2020.

[26] Iglewicz B Hoaglin DC : *How to Detect and Handle Outliers.* ASQC Quality Press;1993.

[27] Kelsy Cabello-Solorzano I Ortigosa de Araujo M Peña LC : The impact of data normalization on the accuracy of machine learning algorithms: A comparative analysis. *18th International Conference on Soft Computing Models in Industrial and Environmental Applications (SOCO 2023).* Springer;2023; pp.344–353. 10.1007/978-3-031-42536-3_33

[28] Goodfellow I Bengio Y Courville A : *Deep Learning.* MIT Press;2016.

[29] Zheng W Hu J : Multivariate Time Series Prediction Based on Temporal Change Information Learning Method. *IEEE Trans. Neural Networks Learn. Syst.* 2021;34:7034–7048. 34982703 10.1109/TNNLS.2021.3137178

[30] Pascanu R Mikolov T Bengio Y : On the difficulty of training recurrent neural networks. *Proceedings of ICML.* 2013;1310–1318. 10.48550/arXiv.1211.5063

[31] Hochreiter S Schmidhuber J : Long short-term memory. *Neural Comput.* 1997;9(8):1735–1780. 10.1162/neco.1997.9.8.1735 9377276

[32] Greff K Srivastava R Koutník J : LSTM: A search space odyssey. *IEEE Trans. Neural Networks Learn. Syst.* 2017;28(10):2222–2232. 27411231 10.48550/arXiv.1503.04069

[33] Gers FA Schmidhuber J Cummins F : Learning to forget: Continual prediction with LSTM. *Neural Comput.* 2000;12(10):2451–2471. 11032042 10.1049/cp:19991218

[34] Ke G Meng Q Finley T : LightGBM: A highly efficient gradient boosting decision tree. *Advances in Neural Information Processing Systems (NeurIPS).* 2017;30:3146–3154. NIPS’17: Proceedings of the 31st International Conference on Neural Information Processing Systems.

[35] Hanif MF Naveed MS Metwaly M : Advancing solar energy forecasting with modified ANN and LightGBM learning algorithms. *AIMS Energy.* 2024;12(2):350–386. 10.3934/energy.2024017

[36] Abbas IT Ghayyib MN : Using sensitivity analysis in linear programming with practical physical applications. *Iraqi Journal of Science.* 2024;907–922. 10.24996/ijs.2024.65.2.27

[37] Abbas IT Aziz AAH : HMPCS–ML Raw Dataset for Solar Power Forecasting (Version 1.0).[Data set]. *Zenodo.* 2025. 10.5281/zenodo.17560035

[38] Abbas IT Aziz AAH : Synthetic datasets for HMPCS–ML-based solar power forecasting. *Zenodo.* 2025. 10.5281/zenodo.17432485

