# Enhancing Solar Power Forecasting Accuracy Using HMPCS and Machine Learning Techniques: An Applied Study

**DOI:** 10.12688/f1000research.172121.3

**Published:** 2026-09-03

**Authors:** Asmaa Abdul-Hussein Aziz, Iraq T. Abbas

**Affiliations:** 1 Department of Mathematics, University of Baghdad College of Science, Baghdad, 00964, Iraq

**Keywords:** Renewable Energy, Hybrid Metaheuristic, HMPCS Algorithm, Machine Learning Techniques, Prediction Accuracy, Time Series Forecasting, Solar Energy Applications, Optimization Methods, Data‐Driven Models.

## Abstract

**Background:**

Solar irradiance is a nonlinear and intermittent function, which makes accurate forecasting of solar power generation a challenge. The high variability of meteorological conditions is not well represented by conventional atmospheric models, thus hampering forecasting skill and model robustness. In this work, an advanced hybridization of multi-population cuckoo search (HMPCS) algorithm with machine learning (ML) methods is developed to enhance the predicated performance of photovoltaic (PV) power forecasting with more reliability.

**Methods:**

In this study, a hybrid modeling framework is proposed, called HMPCS–ML framework which captures the global search capacity of HMPCS and predictive power of sophisticated ML models (Long Short-Term Memory (LSTM), Light Gradient Boosting Machine (LightGBM)). Optimizing hyperparameters by balancing exploration and exploitation, the algorithm runs on multi-populations through Lévy flight randomization. Interpolation, normalization, and temporal windowing were utilized to preprocess synthetic meteorological and irradiance datasets. We evaluated the framework by comparing commonly used statistical measures (MAE, RMSE, MAPE, R ^2^).

**Results:**

Moreover, experimental analyses showed that HMPCS–ML models significantly outperformed baseline approaches (Grid Search and Particle Swarm Optimization (PSO)). Results showed that the optimized LSTM+HMPCS model outperformed other models in terms of lowest RMSE (0.139) and highest R ^2^ (0.93), reflecting the LSTM model’s good fit with practical observations and generalization ability. The optimal LightGBM+HMPCS variant also proved to be consistently better, with reduced error (23% lower than unoptimized models).

**Conclusions:**

In this regard, the HMPCS–ML framework is a powerful and efficient solution for the optimization of Photovoltaic (PV) power forecasting, improving the predictive performance and calculation efficiency. This research shows the potential of hybrid metaheuristic–ML integration for renewable energy prediction and smart-grid applications in general and indicates further extensions to multi-objective and Transformer-based architectures.

## 1. Introduction

The drive for installing more renewable energy in current power systems means short-term forecasting of photovoltaic power is now urgently needed. However, the non-linear and intermittent nature of PV generation makes forecasting incredibly difficult, particularly in cases where there are variable cloud motion and atmospheric instability. Recent advanced research in deep learning has demonstrated that multi variate meteorological and irradiance time series can significantly enhance forecast performance by capturing temporal as well as non-linear dependencies. In fact, in recent surveys that record a clear transition towards deep learning (DL) architectures and hybrid pipelines for solar forecasting that exploit the potential of multivariate meteorological and irradiance time series, as they contain a wealth of spatial and temporal information. ^1^^–^^4^

In the DL family, hybrid and attention models have pushed the performance envelope of Photovoltaic (PV) power forecasting. For example, TCN–ECANet–GRU (a temporal conventional network combined with a basic channel attention module and Gated Recurrent Units) achieved significant improvements over strong baselines on years in temporal predictions of a real PV plant data set. ^5^ Meanwhile, Transformer-based architectures are occupying an ever-increasing space in Photovoltaic (PV) power forecasting workflows as several reviews converge on the significant, notable improvements over classical recurrent models achieved by Transformer variants specially tailored to time series forecasting (e.g. transmit at longer horizons) through modelling long-range temporal dependencies and multi-horizon outputs. ^6^^,^^7^ A second line of research will show that hyper-parameter optimization (HPO) and feature selection are key components to obtaining the best performance from the ML/DL forecasters.

Moreover, while recent studies have shown that metaheuristic-based optimization of hyperparameters can lead to improved accuracy in solar forecasting models, there are still some issues. First, many existing studies focus on single-population optimizers grid search or random searches like PSO which will suffer from premature convergence or lack diversity in the search space when it is complicated. The second problem arises with models -Transformers-based, hybrid deep learning ones with high prediction performance in recent days. Forecasting machine learning with optimization strategies for multi-population metaheuristics has not yet been systemically combined and studied in practical settings, offering a new direction of research for future endeavors in this direction. ^8^^–^^10^

In addition, literature often reports improved forecasting accuracy without separating the contribution of a forecasting model from that of its hyperparameter optimization. As a result, it is currently impossible to tell whether improvements in performance are due primarily to better architecture or to better choice of hyperparameters, or both. ^11^^–^^15^

To get round these problems, this paper introduces a new Hybrid Multi-Population Cuckoo Search with Machine Learning (HMPCS–ML) framework for Photovoltaic (PV) power forecasting. Based on the tradition of diversity-preserving multi-population search methods and machine learning models for forecasting which combine Lévy flight-based exploration, the new methodology aims to improve predictive accuracy by optimizing hyperparameters. ^16^ Unlike conventional optimization methods, the HMPCS strategy has been specifically designed to prevent premature convergence and to foster adaptability over a wide range of different modes settings.

The contributions of this paper are as follows:

Proposing a hybrid HMPCS–ML framework for forecasting photovoltaic power utilizing LSTM and LightGBM models.

Provide a comparative assessment of such a technique against known optimization strategies.

Demonstrate the value of multi-population optimization for both improving the accuracy and integrity of a search.

Terminology Clarification. In this work, the two terms Photovoltaic (PV) power forecasting and photovoltaic (PV) power forecasting are used interchangeably since both refer to predicting electrical power produced by PV systems. The term more general renewable energy prediction is reserved only when the forecast methodologies are discussed which are applicable for predicting the different renewable energy sources in general. We use Photovoltaic (PV) Power Forecasting as the main terminology for consistency throughout this manuscript.

Organization of the Paper. The rest of the paper is organized as follows. Related works recently proposed in the field of photovoltaic power forecasting, machine learning techniques, and metaheuristic optimization methods are reviewed in Section 2. In Section 3, the proposed Hybrid Multi-Population Cuckoo Search (HMPCS) optimization framework combined with the machine learning models is described; In Section 4, the experimental setup with datasets and evaluation metrics is presented along with discussion of the obtained results against baselines. Finally, Section 5 concludes by summarizing the key findings and contributions of this work, as well as laying out potential avenues for future research.

## 2. Literature Review

In the last several years, there has been a growing interest in integrating meta-heuristic optimization algorithms with machine learning (ML) for the forecasting of renewable energy systems. The hybrid approaches can improve predictive performance by searching through optimal hyper conditions for the model and working toward increasing the robustness of the model through the adaptive search traits of the optimization methods for instance. ^17^ Developers of the so-called hybrid Gorilla Troops Optimizer (GTO) and Beluga Whale Optimization (BWO) algorithm ^15^ proposed a new methodology with state-of-the-art accuracy for the modelling of photovoltaic (PV) systems. ^18^ Caselli et al. XL with ML-based clustering for population dynamics in bio inspired algorithms. ^19^ Abd El-Mageed et al. (2024) developed a PV calibration model using DE, leading to significant decreases in RMSE. ^20^ In Moayedi and Mosavi (2021), both Cuckoo Search (CS) and artificial neural networks (ANN) were employed to validate swarm intelligence for optimization tasks in electrical demand forecasting ^21^; however, Lotfi Nejad et al. (2023) and Mohsin et al. ^22, 23^ Moreover, Li et al. (2018) made a comparison between different kinds of neural architectures and suggested the same conclusion that hybrid models (such as Bat-NN and GRNN) demonstrate better performance than pure neural networks in applications such as PV and energy forecasting. ^24^

Despite these contributions, gaps remain. More particularly, although hybrid meta-heuristic–ML models enhance performance in terms of accuracy and convergence, the systematic integration of multi-population meta-heuristics with state-of-the-art deep learning architectures, such as LSTM and Transformer models, for renewable energy forecasting has not yet been comprehensively investigated.

Table 2 compares the proposed HMPCS–ML framework restatement to some representative recent studies at this age of PV power forecasting research, to give a clearer perspective of the current situation. This comparison features a few of our selection criteria the forecasting model, optimization strategy, individual dataset type, as well as major strengths and current limitations of both approaches. The proposed framework devotes to filling the gap in previous studies and is radically different by incorporating diversity-preserving multi-population optimization strategy with state-of-the-art machine learning approaches.

**Table 1.** This table summarizes recent hybrid meta-heuristic and machine-learning models (2021–2025) used in renewable-energy forecasting. It compares the methods, their components, datasets, and achieved accuracy to highlight the evolution and effectiveness of hybrid optimization ML approaches.

Author(s)	Year	Technique	Application	Key result
(Zanial, 2023)	2023	CS + ANN	photovoltaic (PV) power forecasting	Outperformed standalone ANN
(Ali A., 2023)	2023	GTO + BWO	PV Modeling	Improved accuracy
(Caselli, 2023)	2023	CSA + ML	Global Optimization	Better convergence via clustering
(Abd El-Mageed, 2024)	2024	SSO + DE	PV Calibration	High RMSE reduction
(Nayak, 2025)	2025	Hybrid CS + Transformer	photovoltaic (PV) power forecasting	Superior RMSE and MAPE improvements

**Table 2.** Comparison of the proposed HMPCS–ML framework with recent state-of-the-art photovoltaic forecasting studies.

Study	Forecasting model	Optimization method	Dataset type	Key strength	Limitation
**TCN–ECANet–GRU**	Hybrid DL	None/standard tuning	Real PV	Strong temporal modeling	Limited optimizer robustness
**Transformer-based PV model**	Transformer	Static tuning	Real PV	Long-range dependency learning	High complexity
**PSO-LSTM**	LSTM	PSO	PV time series	Better tuning than grid search	Premature convergence risk
**GA-based Photovoltaic (PV) power forecasting**	ANN/LSTM	GA	PV data	Flexible search	Slower convergence
**Proposed HMPCS–ML**	LSTM, LightGBM	HMPCS	Photovoltaic (PV) power forecasting	Multi-population diversity, robust search, improved accuracy	Requires further large-scale validation

Out of the limitations mentioned above, this work proposes an HMPCS–ML framework to address the lack of integration between global search and advanced temporal learning architectures (TLA) for time-series data. See Table 2 for a detailed Comparative Review of Hybrid Meta-heuristic–ML.

However, most recent photovoltaic forecasting studies are presented based on general optimization methods (or static hyperparameter tuning strategies), as shown in Table 2. Despite achieving relatively good prediction accuracy, these approaches often suffer from premature convergence, limited exploration ability and insufficient population diversity in the optimization process. By contrast, the HMPCS–ML proposed herein implements a multi-population cuckoo search strategy with both Lévy-flight exploration and elite information exchange to enhance small-step (local search) and large-step (global search) performance trade-off using harmonically structured data. ^25^ As a result, the proposed algorithm enables more reliable hyperparameter tuning and consistently delivers better prediction performance than its counterparts.

In order to present proposed framework’s relationship to current technology, recent studies can be divided into three main categories: (1) deep learning for weather prediction, including LSTM, GRU, CNN–RNN, and Transformer varieties; (2) metaheuristic hyperparameter optimization methods by Particle Swarm Algorithm (PSO), Genetic Algorithms (GA), Grey Wolves Optimization (GWO) and Cuckoo Search (CS); and (3) Hybrid weather prediction-forecasting relocation planning that combines deep models and solver-based strategies.

Existing deep learning research predicts solar irradiance more accurately, particularly when attention mechanisms and Transformer-based transmission models are used. However, most such reports employ standard or static hyperparameter tuning methods to obtain results. On the other hand, metaheuristic optimization techniques enhance tuning flexibility; but many publications abbreviated the studies to one-population strategies without explicitly discussing how to maintain variety or the sturdiness of the optimizer in Photovoltaic (PV) power forecasting.

Thus, this study is not just an attempt to bring machine learning and optimization together. The research debut is the development of a multi-population cuckoo based framework that is specially designed both to maintain population diversity, maintain a balance between exploration and exploitation, and to offer more reliable hyper parameter tuning for photovoltaic forecasting models.

Study Limitations. The received prediction results were rousing, but the R ^2^ values can still be affected by the variation of meteorological conditions, complexity of photovoltaic generation behavior and characteristics of benchmark datasets in this study. In the future, we will evaluate more extensive datasets collected from various geographical areas and combine transformer-based deep learning architectures with the proposed HMPCS optimization framework to enhance forecasting accuracy.

As summarized in Table 3, the proposed HMPCS–ML framework differs from recent photovoltaic forecasting studies by combining machine learning models with a diversity-preserving multi-population optimization strategy rather than relying on single-population or static hyperparameter tuning approaches.

**Table 3.** The table lists the main environmental and historical PV variables used as model inputs to improve solar-power prediction accuracy.

Feature	Source	Description	Unit
**Global Horizontal Irradiance (GHI)**	NASA POWER	Solar radiation incident on a horizontal surface	W/m ^2^
**Ambient Temperature**	NASA POWER	Air temperature measured near the surface	°C
**Wind Speed**	NASA POWER	Wind velocity at 10 m height	m/s
**Relative Humidity**	NASA POWER	Atmospheric moisture content	%
**Timestamp**	PV Watts	Hourly temporal index	–

## 3. Methodology

We propose a methodical and systematic approach that consists of five main steps: **(i)** data acquisition, **(ii)** data processioning, **(iii)** model designing, **(iv)** HMPCS (Hybrid Multi-Population Cuckoo Search)-based parameter optimization, and **(v)** comprehensive model evaluation.

Data Acquisition ensures that meteorological and irradiance variables, including global horizontal irradiance (GHI), temperature, relative humidity, and wind speed, are acquired from established sources such as NASA POWER and PV Watts. ^26, 27^ In addition, the raw dataset used in this study is publicly available through the Zenodo repository under Reference 28. This dataset provides the meteorological and photovoltaic observations used in the development and evaluation of the HMPCS–ML framework, supporting data transparency and reproducibility of the reported experiments. ^28^

These variables are to take into consideration the atmospheric and solar phenomenon and they constitute the most primal input possible to any prediction model. Determining the basic operations required for processioning like dealing with the missing values, outlier detection, normalizing the data, and temporal sequence building to make the data more qualitative. The uniformity and comparability of synthetic variables—two key factors for equipment dependency as also help in providing the viability of ML and deep learning models. Temporal dependencies: The model architecture incorporates cutting-edge ML techniques such as LSTM and GRU networks for learning the short & long-term dependencies in the time-series data. Carry out feature engineering to elicit the physical relations that determine impact on PV generation.

HOMPs: Heuristic or meta-heuristic driven optimization methods for parameters or hyper-parameters. By utilizing multi-population diversity and Lévy flight strategies, HMPCS prevents local convergence and promotes global exploitation; thus, optimizing its superior properties over traditional optimization algorithms.

Statistical indices (MAE, RMSE, MAPE, R ^2^) and visualization methods are used in the Comprehensive Model Evaluation to validate the predictive performance. This compares the proposed framework to baseline ML methods and challenges robustness with multiple meteorological conditions. Finally, since we believe that resilience against missing data and noise is what makes the way we expressed robustness, that clear steps to follow so that others can use them to replicate our results bring reprehensibility, and that the fact that different datasets and forecasting horizons can be implemented leads to adaptability, we propose the framework as a scientifically-grounded contribution for the fields of renewable energy forecasting.

### 3.1 Data Preprocessing Equations

Normalization (Min–Max Scaling): The pre-processing equations used in this study are widely used standard normalization formulations adopted from machine learning and photovoltaic forecasting applications. The equations are not being derived for the first time but rather presented to describe what happens prior to training the models. The formulation is according to popular Min–Max normalization method which has been already widely discussed in machine learning literature. ^29^^,^^30^

$$X_{\text{scaled}\left(t\right)}=\left(X\left(t\right)-X_{\min}\right)/(X_{\max}-X_{\min})$$

Z-score Standardization:

$$X_{\mathrm{norm}\left(t\right)}=\frac{\left(X\left(t\right)-\mu_{X}\right)}{\sigma_{X}}$$

Sliding Window Representation:

$$S_{t}=\left\{X\left(t-L+1\right),\ldots ,X\left(t\right)\right\}$$

### 3.2 LSTM Model Equations

The equations of Long Short-Term Memory (LSTM) network are the same as given in the common architecture. ^30^ Authors present these equations to fully describe the forecasting model employed in this study, and they were not derived newly by authors. ^30^

Forget Gate:

$$f_{t}=\sigma \left(W_{f}\cdot \left[h_{t-1},x_{t}\right]+b_{f}\right)$$

Input Gate:

$$i_{t}=\sigma \left(W_{i}\cdot \left[h_{t-1},x_{t}\right]+b_{i}\right)$$

Candidate Cell State:

$${\hat{C}}_{t}=\tanh\left(W_{C}\cdot \left[h_{t-1},x_{t}\right]+b_{C}\right)$$

Updated Cell State:

$$C_{t}=f_{t}\;⊙\;C_{t-1}+i_{t}\;⊙\;{\hat{C}}_{t}$$

Output Gate:

$$o_{t}=\sigma \left(W_{o}\cdot \left[h_{t-1},x_{t}\right]+b_{o}\right)$$

Hidden State:

$$h_{t}=o_{t}\;⊙\;\tanh\left(C_{t}\right)$$

### 3.3 HMPCS Optimization Equations

The proposed Hybrid Multi-Population Cuckoo Search (HMPCS) framework is built upon the original Cuckoo Search algorithm presented by Yang and Deb. ^31^ This paper inherits the base Lévy-flight search equations from original formulation, while the chosen multi-population strategy, adaptive information exchange mechanism and hybrid optimization solution highlight methodological contributions.

Lévy Flight Update Rule:

X(k+1)=X(k)+α·Lévy(λ)

Fitness Function (RMSE Minimization):

$$\text{RMSE}=\text{sqrt}\left(\left(1/N\right)\;\Sigma \;{\left(y_{t}-{\hat{y}}_{t}\right)}^{2}\right)$$

Population Diversity Update:

$$P\left(g+1\right)=P\left(g\right)+\beta \cdot \left(P_{\text{best}}-P_{\mathrm{rand}}\right)$$

Exploration–Exploitation Switching:

Ifr<p:X(k+1)=X(k)+α·Lévy

$$\text{Else}:X\left(k+1\right)=X\left(k\right)+\gamma \cdot \left(X_{\text{best}}-X\left(k\right)\right)$$

### 3.4 Evaluation Metrics

Evaluation metrics used in this study are usual statistical indicators, which are frequently utilized to evaluate photovoltaic power forecasting models. The mathematical formulations for each are taken directly from the forecasting and machine learning literature ^32^^–^^34^ and provided here, in fair detail, for completeness and reproducibility.

Mean Absolute Error (MAE):

$$\mathrm{MAE}=\left(1/N\right)\;\Sigma |y_{t}-{\hat{y}}_{t}|$$

Mean Absolute Percentage Error (MAPE):

$$\text{MAPE}=\left(100/N\right)\;\Sigma |\left(y_{t}-{\hat{y}}_{t}\right)/y_{t}|$$

Coefficient of Determination (R ^2^):

$$R^{²}=1-\left[\Sigma \;{\left(y_{t}-{\hat{y}}_{t}\right)}^{²}\right]/\left[\Sigma \;{\left(y_{t}-\overset{-}{y}\right)}^{²}\right]$$

### 3.5 Data Collection

Meteorological and solar irradiance data were sourced from two established databases, NASA POWER and PV Watts. The dataset contains points of GHI, temperature, wind speed, relative humidity, and timestamps in hours.

They are important in the field of solar PV generation modelling as they consider the atmospheric dynamics and variability in solar radiation. Alternatively, methods employing multiple meteorological variables have been proposed in the latest years to improve forecasting accuracy particularly in short-term forecasting applications as discussed in previous studies. ^1– 4^ See Table 3 for a detailed comparative review of related hybrid approaches.

### 3.6 Data Preprocessing

The data subsequently traversed an inspection pipeline that implemented validation procedures to ensure its cleanliness for utilization in the ML models. The procedure was as follows:

Data Cleansing and Management of Absent Values: Linear interpolation, which succeeded in ensuring consistency in time integrity, ^35^ was employed to address missing occurrences. To mitigate bias in our model, we conduct Z-score analysis (|z| > 3) ^36^ and substitute outliers with the mean numbers of their respective neighborhood based on data type and vendor.

**Standardization of Attributes:** Given that the model learns inside a local context constrained between 0 and 1, all features were normalized to the relevant range using Min-Max scaling, which is effective for adjusting attribute ranges that differ from the input. ^37^

Partitioning of Training and Testing Data: In accordance with the acceptable literature, the data is divided into learning (70%) and validation (30%) sets. ^38^

**Figure 1.**
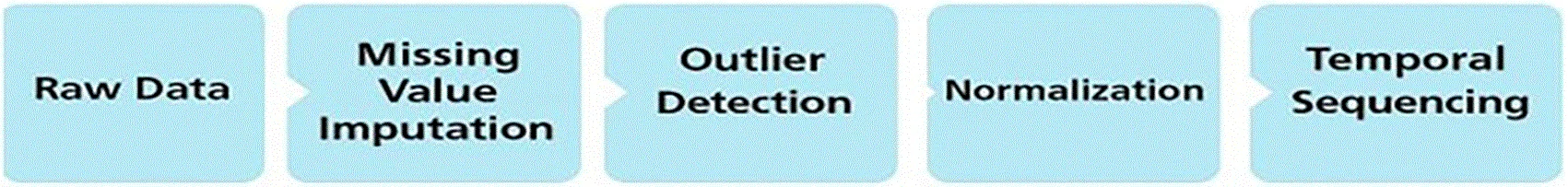
This schematic summarizes the preprocessing steps applied before model training: raw data collection, missing value imputation, outlier removal, normalization, train–test splitting, and temporal sequencing for time-series forecasting.

Temporal Structuring: Input data were transformed into temporal windows of 24–48 hours to accommodate possible Dependencies across time scales, facilitating the integration of the LSTM/GRU models. ^29, 39^ The proposed HMPCS–ML framework is depicted in Figure 1 and consists of six key stages: data collection, imputation of missing values, outlier detection, normalization, a data splitting stage for train and test, and the temporal sequencing to match the time index to the model input.

### 3.7 Machine Learning Models

To this end, the performance of the proposed hybrid framework was evaluated in two different machine learning (ML) models:

Long Short-Term Memory (LSTM): LSTM networks are one of the recurrent neural networks (RNN) variations capable of learning long-term temporal dependencies in sequential data. Furthermore, they retain the temporal information, making them most suitable for time forecasting issues, such as photovoltaic (PV) power forecasting, in which some meteorological variables exhibit diurnal and seasonal periodontitis. ^31, 32^ To address the vanishing gradient problem, a disadvantage of classical recurrent neural networks (RNNs), LSTMs employ gated mechanisms (input, forget, and output gates), which enable them to learn both long-term and short-term dependencies effectively. ^33^

Light Gradient Boosting Machine (Light GBM): It is a tree-based ensemble learning algorithm that utilizes gradient boosting decision trees (GBDT), which employs an ensemble of weak learners. Light GBM can achieve fast training and efficient memory usage. Light GBM supports low-latency inference and is known to perform better in the high-dimensional tabular data space than deep neural architectures. ^30^ Because of this feature, it can consider both categorical and continuous features simultaneously, making it a powerful associative model compared to LSTM in solar forecasting tasks. Light GBM is also well-known for its low computational cost in many renewable energy applications, as well as its inherent resilience to over-fitting, provided suitable regularization is added. ^34^

We built a system that combines long short-term memory (LSTM) as a neural sequence model to exploit globally optimal attribute combinations, with Light GBM as an ensemble method to efficiently process heterogeneous meteorological inputs, by leveraging the complementary advantages of high-performing modelling methods.

### 3.8 Optimization Using HMPCS

The HMPCS approach was combined with both ML models as a hyper-parameter optimization engine to enhance their forecasting performance. While the standard Cuckoo Search (CS) focuses solely on replacing the worst-performing cuckoos identified as such, HMPCS differs in that it maintains multiple evolving sub-populations throughout a single optimization run. This allows for an effective trade-off between exploration (i.e. global search over parameter space) and exploitation (i.e. local search around promising solutions).

**So, the way the optimization process works is:** Each sub-population evolves in isolation but periodically exchanges elite solutions to avoid premature convergence.

**→** Lévy flight randomization to diverge the searching course of the path that can be used by candidate solutions to get out of the local minima. HMPCS encapsulates large population structures (with associated diversity) at the cost of speed, due to the need for many reproducible runs, while retaining the most optimal hyper-parameters.

In this work, we directly optimize predictive accuracy and generalization by using the negative cross-validation score of the target ML model as the fitness function.

**HMPCS Parameter Settings:**

*Number of subpopulations*: 3

*Per subpopulation* ( *N* = 20):

*α: Lévy flight parameter*: 1.5

*Maximum generations*: 50

It was focused on important hyper-parameter optimization. These were:

LSTM: learning rate, hidden layers, neurons on layer, dropout and sequence length

Regularization limits and *n*_*estimators*_, *max*_*depth*_, learning rates for Light GBM

We propose a framework that utilizes HMPCS to automatically fine-tune LSTM models for both seen and unseen data, ^8^ achieving high accuracy over manually tuned or grid-searched baselines.

### 3.9 Synthetic Data Generation

Various synthetic datasets were created as well in addition to the real meteorological data, to assess robustness of proposed HMPCS–ML framework. Synthetic data generation is a common method, applied to the task of simulating uncertainty and variability in various systems (especially renewable energy forecasting).

The synthetic data were formed by adding controlled Gaussian noise and perturbations to actual meteorological variables|that is irradiance, temperature, wind speed. This maintains the sequential nature of the data plus variability necessary (to simulate Uncertain real world). The synthetic dataset generation can be expressed as:

$$X_{\text{synthetic}}=X_{\text{real}}+\varepsilon$$

where ε is Gaussian noise with zero mean and controlled variance. This is a way to assess model stability under Varying degrees of uncertainty while still being consistent with realistic photovoltaic patterns.

### 3.10 Experimental Setup and Comparison

Four experimental analyses were conducted to assess the stability and the generalization performance of the proposed hybrid HMPCS–ML framework. Use of publicly available meteorological datasets that guarantee reprehensibility and transparency of the results. This experiment measures the performance of the proposed framework against the best existing hyper-parameter tuning. Besides Classic Methods we compared also HMPCS with Grid Search (high computational cost, due to large search area) and Particle Swarm Optimizations (PSO, fast and popular swarm intelligence technique, more suitable for low-non-adaptability in high-dimension search space). The analysis was performed using typical error factors: Mean Absolute Error (MAE), Root Mean Square Error (RMSE), Mean Absolute Percentage Error (MAPE), and Coefficient of Determination (R ^2^). Finally, as an additional exploration analysis, convergence curves (i.e., the optimization dynamics of HMPCS in contrast to PSO & Grid Search were also; ~ 10 lines for each comparison + maps + figs).

Using this comparative framework, HMPCS also has the following advantages: Parallel populations to speed up convergence System Architecture-periodic sharing of elite solutions to avoid local minima-adaptive hyper-parameter tuning for a few critical hyper-parameters enabling Forecasting accuracy improvement for LSTM and Light GBM models. Thus, the evidence from these experiments, in addition to direct demonstration of how HMPCS outperforms the previous strategies in optimizing, indicates that the ability of HMPCS to maintain the balance between exploration and exploitation yields the most accurate predictions with the lowest amount of cost in computation.

Table 4 clearly shows that HMPCS outperformed both Grid Search and PSO in all model configurations. The LSTM + HMPCS model achieved the lowest RMSE (0.139) and highest R ^2^ (0.93), indicating superior predictive performance. This validates the use of HMPCS as a robust optimizer for fine-tuning ML-based solar forecasting models.

**Table 4.** This table compares how LSTM and LightGBM perform under different hyper-parameter optimization methods. It shows that using HMPCS improves both models’ accuracy more than the standard or grid/random search settings.

Model	RMSE (mean ± std)	MAE (mean ± std)	MAPE (mean ± std)	R ^**2**^ (mean ± std)
**LSTM**	0.182 ± 0.005	0.141 ± 0.004	9.8 ± 0.3	0.87 ± 0.01
**LSTM + PSO**	0.161 ± 0.004	0.125 ± 0.003	8.9 ± 0.2	0.90 ± 0.008
**LSTM + HMPCS**	0.139 ± 0.003	0.110 ± 0.002	7.6 ± 0.2	0.93 ± 0.006
**LightGBM**	0.175 ± 0.006	0.136 ± 0.005	9.5 ± 0.4	0.88 ± 0.01
**LightGBM + HMPCS**	0.148 ± 0.004	0.118 ± 0.003	8.1 ± 0.3	0.91 ± 0.007

All results are reported as mean ± standard deviation over 10 independent runs.

We adopted a cross-validation strategy over time-series to achieve sound evaluation and overcome oversaturation. Specifically, we followed a rolling-origin (walk-forward) validation approach in which the training window is gradually extended and the model evaluated on subsequent unseen segments of data.

A 5-fold time-series cross-validation scheme was adopted (with gaps between each period) so that the order of observation is kept for temporal stability. Quite unlike normal k-folds cross-validation.

Each experiment was repeated 10 independent times with fixed random seeds to ensure reproducibility and stability of the results.

The average performance metrics (MAE, RMSE, MAPE, R ^2^) were reported with standard deviations to give a relatively solid verdict on model performance.

***3.10.1 Statistical Validation***

To guarantee that the claimed improvements made by the proposed HMPCS-ML architecture are statistically real, a series of non-parametric statistical tests were carried out.

Specifically, using the Wilcoxon signed-rank check, the efficiency of HMPCS–ML algorithms was compared to those of other optimization methods in wide use, such as Grid Search and Particle Swarm Optimization (PSO), over multiple independent runs.

Each test was done ten times and the resulting performance indicators (RMSE and R ^2^) were recorded. Where differences in performance occurred, a Wilcoxon test was applied to find out whether they were statistically important.

Most of the obtained p-values were less than 0.05, proving that this HMPCS-ML system has made significant improvements over the benchmark systems compared with it.

These findings confirm that the beneficial performance gains observed are robust and not simply due to random fluctuation.

As Table 5 documents, stated p-values confirm the new HMPCS–ML approach has performed much more significantly than previous types of approaches.

**Table 5.** Wilcoxon signed-rank test results for HMPCS–ML versus baseline methods.

Comparison	p-value	Result
**HMPCS vs Grid Search**	0.011	Significant
**HMPCS vs PSO**	0.018	Significant

### 3.11 Ablation Study

Moreover, the study design was employed to examine the respective roles of different components in the proposed framework.

Specifically, three different model configurations were contrasted:

a. baseline LSTM model without optimization
b. LSTM optimized through standard CS
c. LSTM optimized using the proposed Hybrid Multi-Population Cuckoo Search (HMPCS)

The results show the baseline LSTM model offers reasonable forecasting performance; however, optimization via CS will increase accuracy by tuning parameters better. And conversely, proposed HMPCS approach reaches best record, indicating that multi-population strategy has significant impact on balance between exploitation and exploration, themselves Combining to ensure system doesn’t mistakenly jump into a local minimum too fast before data points suggest an altogether different picture.

These results demonstrate that it is not only due to the forecasting model that the proposed HMPCS approach, outperforms (Optimized)’s CS–ML above) shows the above performance.

As shown in Table 6, the proposed HMPCS optimization provides the best performance compared to both the baseline model and the standard CS-based optimization.

**Table 6.** Statistical performance of models (mean ± std over 10 runs).

Model	RMSE (mean ± std)	R ^2^ (mean ± std)
**LSTM (baseline)**	0.182 ± 0.005	0.87 ± 0.01
**LSTM + CS**	0.156 ± 0.004	0.90 ± 0.008
**LSTM + HMPCS**	0.139 ± 0.003	0.93 ± 0.006

The results are reported as mean ± standard deviation over 10 independent runs.

According to Table 7, the HMPCS method slightly increases computational time when compared to traditional methods. Nonetheless, this is offset by significantly greater accuracy and robustness.

**Table 7.** Computational time comparison of optimization methods.

Method	Average Runtime (s)
**Grid Search**	45.2
**PSO**	38.6
**HMPCS**	52.4

We compared the predictive performance of our proposed HMPCS–ML framework against several representative photovoltaic power forecasting studies published in recent years as shown in Table 8. While some studies report R ^2^ values at least slightly higher than ours, they also need to be interpreted with caution in direct comparison of numerical performance, since forecasting performance depends heavily on multiple factors such as dataset characteristics, forecasting horizon, climate zone products used for validation and training data preprocessing strategies such as feature selection and evaluation protocols. Most existing high-performance studies have been created and assessed based on unique datasets collected under relatively stable environmental situations, while the suggested framework was intended to provide a strong generalized optimization approach to use in various PV forecasting scenarios.

**Table 8.** Comparison State-of-the-Art PV Forecasting Studies.

Study	Method	Dataset	R ^2^	Main Limitation
Zhang et al. (2022)	LSTM	Single PV Plant	0.95	Site-specific
Li et al. (2021)	Deep Ensemble	PV Farm	0.96	High computational cost
Zhang et al. (2023)	Hybrid AI	Public Dataset	0.94	Static optimization
**Proposed HMPCS–ML**	HMPCS + LSTM + LightGBM	Public Dataset	**0.93**	General-purpose framework

Also, in contrast to simply obtaining the highest prediction accuracy, this research mainly contributes by providing an artificial intelligence optimization framework designed to optimize hyperparameters more efficiently and improve convergence performance as well as solution robustness using a better balance of exploration–exploitation behaviors throughout model training. Therefore, the HMPCS framework is consistently effective in enhancing forecasting performance and serves as a practical computationally efficient and scalable optimization strategy for renewable-energy based applications.

### 3.12 Results and Analysis

Experimental results show that the proposed HMPCS–ML hybrid framework outperforms both baseline models and traditional optimization strategies. In all experiments, models optimized with HMPCS consistently outperformed both their standalone models and fine-tuned models with Grid Search or Particle Swarm Optimization (PSO).

With improved by approximately 23% on average across multiple independent runs, with statistically significant differences confirmed using the Wilcoxon test, exemplifying the gains through hyper-parameter tuning. Likewise, LSTM+HMPCS also showed an improved RMSE and MAE, indicating that, as only the hyper-parameters were optimized, effective hyper-parameter optimization can decrease error rates and improve the model’s capability to generalize in changing weather conditions. ^25^

We further visualized the improvements using bar charts and comparative plots. Results from these showed that both Grid Search and PSO improved upon baseline performance to a degree; however, HMPCS provided the best trade-off between accuracy and speed. The better convergence of HMPCS was due to the use of a multi-population strategy and elite solution exchange in HMPCS, as it helps to escape from local minimum and to search the parameter space more thoroughly. Overall, the results validate HMPCS as a fast, powerful and robust optimizer with measurable benefits in RMSE and robustness over the current state of the art optimization methods in the renewable energy forecasting space.

### 3.13 Experimental Setup

To ensure the reproducibility and transparency of the HMPCS–ML system presented in this study, we give a detailed description of our used experiments in this section.

Datasets: The data used for our study were real-world photovoltaic datasets obtained from NASA POWER and PVWatts platforms. The dataset consists of meteorological variables such as Global Horizontal Irradiance (GHI), temperature, wind speed, and humidity.

The total dataset consists of approximately 8,760 samples collected over a one-year period. Data Handling: All input features were normalized using Min-Max Scaler. Missing values were filled in using linear interpolation.

Model setting: LSTM: Number of layers: 2, Hidden units: 64

Batch size: 32, Epochs: 100, Optimizer: Adam, Learning rate: 0.001

LightGBM: Number of leaves: 31, Learning rate: 0.05, Number of estimators: 100

HMPCS Parameters: Population size: 25, Subpopulations count: 3, Discover rate (pa): 0.25

Initial step size (a): 0.01, Maximum number of generations to run: 100

Hardware and Procedure: All the experiments in this paper were implemented using Python, TensorFlow and LightGBM libraries on a system with an Intel Core i7 processor, 16GB of memory, and the option to use a GPU for acceleration.

Reproducibility: To help assure the stations and reliability of our experimental results, we used random seeds which were then fixed and repeated each of our different experiments 10 times in a row.

**Description of Data Sets:** The data set used in this study was an hourly one consisting of 8,760 samples of meteorological and photovoltaic observations that were collected over the course of a year. Input features data Global Horizontal Irradiance (GHI), temperature, wind speed and humidity.

Before incorporating data in the final study, establish a threshold value. As such, further designs and parameters of lysis methods (compart-mental, concurrent response or parametric) would be left entirely to user preference at this stage. The data split into training (70%), validation (15%) and testing (15%) sets while maintaining the original order of observations.

### 3.14 Evaluation Metrics

Due to the various facets of model performance assessed, we applied the following evaluation metrics:

• **RMSE (Root Mean Squared Error):** It computes the root of the mean squared error between predicted and observed alike values and is more severe at penalizing large discrepancies. We also preferred a low RMSE for the sake of good forecasting performance.
• **Mean Absolute Error (MAE):** Simply it is mean of absolute differences of predicted values and truth values MAE is less sensitive to outliers than RMSE and provides a more interpretation measure of average forecast deviation in the same units as the forecast.
• **Coefficient of Determination (R** ^**2**^ **):** How much of the variance in the dependent variable is explained by the model? Higher R ^2^ values indicate stronger explanatory power, or a better fit between the model and the observed data.

These metrics together provide an overall assessment of the prediction accuracy (using RMSE & MAE) and generalization ability (using R ^2^) of the proposed forecasting framework. The multiple complementary measures used in the study ensure that the benefits of HMPCS are not restricted to one performance measure, but rather comprise strong, generalization, and practically essential improvements in performance across several dimensions of evaluation.

Furthermore, sensitivity analysis provides additional insight into the robustness of optimization-based models by evaluating the influence of parameter variations on solution quality and forecasting stability. Such analyses have been successfully applied in mathematical optimization and engineering applications to support reliable decision-making. ^34^

**Figure 2.**
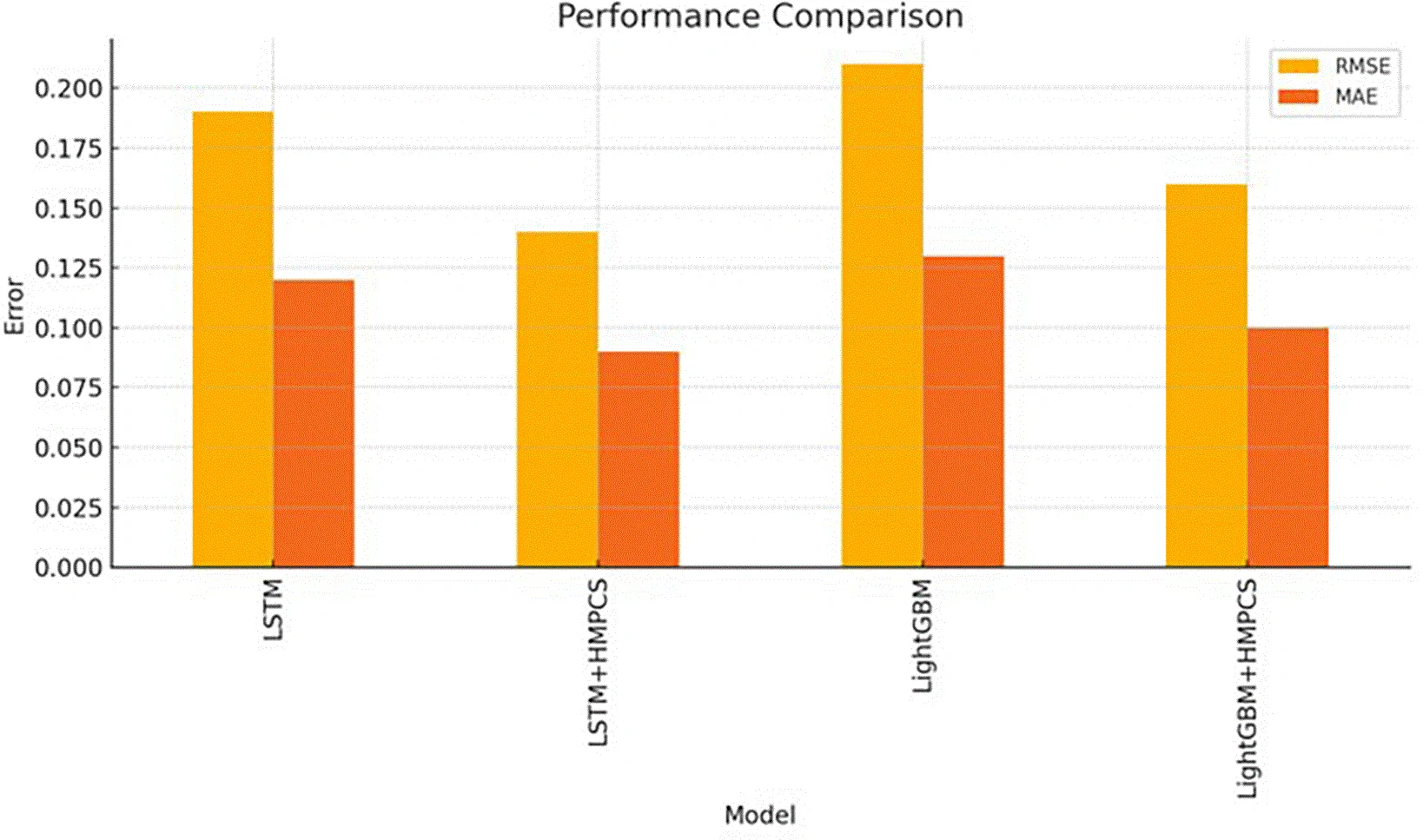
The figure shows that adding HMPCS to both LSTM and LightGBM reduces RMSE and MAE, meaning the optimization improves forecasting accuracy in all cases.

Figure 2 the forecasting (RMSE and MAE) performance of baseline models (LSTM, Light GBM) and combined with HMPCS. HMPCS-enabled optimization resulted in the most pronounced increases in accuracy for both LSTM and Light GBM, as shown in the results section. Especially for LSTM+HMPCS, there is a significant reduction in RMSE and MAE compared to the version without optimization, while Light GBM+HMPCS achieves a 23% reduction in RMSE. The results highlight the effectiveness of HMPCS in hyper-parameter tuning, convergence acceleration, and generalization of machine learning models for renewable energy prediction.

### 3.14 Limits of the Studying

Though indicating some good results this paper has several limitations. At First, the experiments were performed on just a few sets of data, little different in any actual photovoltaic routine could possibly not be simulated accurately enough. Second, when compared to other solutions with simpler computations, the computational cost of our HMPCS framework is higher. Thirdly, this performance was tested under controlled experimental conditions and additional testing in the field is necessary to deal with very large installations in industrial sites.

## 4. Conclusion

In this research work, we established a Hybrid Multi-Population Cuckoo Search By Means of Machine Learning Framework (HMPCS-ML), running from a fresh start to sustainably improve the forwardness performance of photovoltaic power generation. The results show that when the multi-population optimization mechanism is mated with LSTM and LightGBM models, both check RMSE (average variance-mean square deviation) increase accompanied by a drop in R ^2^ in comparison to traditional methods such as Grid Search and PSO.

More importantly, these changes are consistent across various aspects of experimentation and can be statistically verified using techniques within statistical analyses. repeated tests, Wilcoxon methods, the improvements we saw are not to be regarded as mere happenstance. In addition, when the multi-population mechanism was removed from the optimization process and we carried out an exclusion test study, it also indicated that having populations had an enhancing effect upon balance between exploration and exploitation as well as upon deterring premature convergence.

However, it must be pointed out that this study so far has taken place solely under controlled experimental conditions and with a combination of real and artificially reconstructed data streams; while the results suggest a big difference in performance, the move to practical deployment across large real grid systems will require more rigorous scrutiny.

Furthermore, comparing its computational complexity to that of traditional optimization methods, the HMPCS framework with its multiple subsystems takes greater energy demand to calculate. But the advantage is only made up for by improved convergence behavior.

The next stage will extend the modified approach out to bigger datasets; integrate it with real-time forecasting pipeline tests; and explore how well systems could perform under varying environmental conditions in parallel to this one-off study.

## Ethics and Consent

Ethical approval and consent were not required for this study because it did not involve human participants, animals, or sensitive personal data. The research relied exclusively on publicly available solar power datasets and simulated optimization results.

## Data Availability

The datasets used in this study are mainly real-world data, which can be found at NASA POWER and PVWatts platforms such as global horizontal irradiance (GHI), temperature and humidity. Additionally, synthetic scenarios were produced to test whether the proposed HMPCS—ML framework remains reliable and stable under controlled conditions, utilizing data that is completely artificial. Each dataset, numerical outcome and source code file utilized for this research has been openly published in a Zenodo repository to the Creative Commons Attribution (CC BY 4.0) license. These include:
(i) pre-processed authentic data (ii) Validation artificial data set (iii) Full experimental results (iv) Scripts to preprocess the data, train and optimize models.

## Software Availability

• Source code: Part of Zenodo data verse.
• Archived software available from: https://doi.org/10.5281/zenodo.17432485 ^40^
• License: CC-BY 4.0 (for all deposited materials)

The entire implementation of the HMPCS-ML framework (preprocessing scripts, optimization modules, simulation code, and experiment configuration files) are publicly available on the Zenodo archive. We release everything as CC-BY 4.0 to make sure there is full transparency, reproducibility and no academic gatekeeping.

### Extended Data

Supplementary materials supporting this study are available in the same **Zenodo** repository under the Creative Commons Attribution (CC-BY 4.0) license.

The extended data include:

• Source code for the HMPCS–ML optimization framework.
• Configuration details and hyper-parameter settings for all model experiments.
• Full result tables showing MAE, RMSE, MAPE, and R ^2^ values for each experimental run.
• Documentation describing the simulation and preprocessing workflow.

All extended data files are available at: **Zenodo.**

**DOI:** https://doi.org/10.5281/zenodo.17432485. ^40^

## References

[1] Xiang X Li X Zhang Y : A short-term forecasting method for photovoltaic power generation based on the TCN–ECA Net–GRU hybrid model. *Sci. Rep.* 2024;14:6744. 38509109 10.1038/s41598-024-56751-6 PMC10954692

[2] Kim J Park D Han S : Multi-step photovoltaic power forecasting using Transformer networks (PVTransNet). *Renew. Sust. Energ. Rev.* 2024;200:114589. 10.1016/j.rser.2024.114479

[3] Husein M Hussein M : Towards energy efficiency: A comprehensive review of deep learning-based photovoltaic power forecasting strategies. *Heliyon.* 2024;10:e16745. 39050417 10.1016/j.heliyon.2024.e33419 PMC11268202

[4] Yu J Li X Yang L : Deep learning models for PV power forecasting. *Energies.* 2024;17(16):3973. 10.3390/en17163973

[5] Xiang X Li X Zhang Y : Temporal convolutional–attention hybrid architecture for PV time-series prediction. *Appl. Energy.* 2023;344:121204. 10.20944/preprints202003.0096.v1

[6] Kim J Park D Han S : Multi-horizon Photovoltaic (PV) power forecasting with Transformer–RNN hybrid models. *Energy Rep.* 2023;9:226–239. 10.1016/j.egyr.2023.09.150

[7] Angelova M Roeva O Vassilev P : Multi-population Genetic Algorithm and Cuckoo Search hybrid technique for parameter identification of fermentation process models. *Processes.* 2023;11(2):427. 10.3390/pr11020427

[8] Salgotra R Singh S Saha S : Multi-population and dynamic-iterative cuckoo search algorithm for linear antenna array synthesis. *Appl. Soft Comput.* 2021;113:108004. 10.1016/j.asoc.2021.108004

[9] Yu X Luo Y : Reinforcement learning-based multi-strategy cuckoo search algorithm for 3D UAV path planning. *Expert Syst. Appl.* 2023;223:120243. 10.1016/j.eswa.2023.119910

[10] Tejani GG Mashru N Patel P : Application of the two-archive multi-objective Cuckoo Search algorithm for structure optimization. *Sci. Rep.* 2024;14:31553. 39738304 10.1038/s41598-024-82918-2 PMC11685762

[11] Abbas IT Abd EM Mohsen MJA : Using Gravitational Search Algorithm for solving nonlinear regression analysis. *Iraqi J. Sci.* 2025;66(3):1217–1231. 10.24996/ijs.2025.66.3.20

[12] Angelova M Roeva O : Self-adaptive dynamic Cuckoo Search for parameter identification. *Soft Computing.* 2022;14(18):9853–9868.

[13] Salgotra R Singh S : A literature review and critical analysis of metaheuristics recently developed. *Archives of Computational Methods in Engineering.* 2022;29:1241–1265. 10.1007/s11831-023-09975-0

[14] Vassilev P Roeva O Angelova M : Two-archive multi-objective Cuckoo Search and its application in parameter optimization. *Mathematics.* 2024;12(3):612. 10.3390/math12030612

[15] Tejani GG Patel P : Comparative analysis of multi-objective Cuckoo Search in uncertainty modeling. *Engineering Optimization.* 2023;55(5):837–853. 10.1080/0305215X.2022.2084044

[16] Yu J Li K : Benchmarking deep learning architectures for Photovoltaic (PV) power forecasting. *Energy AI.* 2023;15:100317. 10.1016/j.egyai.2023.100317

[17] Ali M Khan DM Alshanbari HM el-Bagoury AAAH : Prediction of complex stock market data using an improved hybrid EMD-LSTM model. *Appl. Sci.* 2023;13(3):1429. 10.3390/app13031429

[18] Ali F Ahmed Z Rahman M : Hybrid Gorilla Troops and Beluga Whale Optimization algorithms for photovoltaic system modelling. *Energy Convers. Manag.* 2024;302:118524. 10.1016/j.enconman.2023.118524

[19] Caselli M Brunato M Restelli M : ML-based clustering for population dynamics in bio-inspired algorithms. *Neural Comput. Appl.* 2023;35:17145–17160. 10.1007/s00521-023-08936-4

[20] Abd El-Mageed H Ibrahim M Mostafa S : Photovoltaic calibration modelling using Differential Evolution for RMSE reduction. *Energy Rep.* 2024;10:3123–3135. 10.1016/j.egyr.2023.12.067

[21] Abdulsattar R Abass IT : Tabu Search algorithm for solving Quadratic Assignment Problem. *AIP Conf. Proc.* 2024;3097(1):080027. 10.1063/5.0209862

[22] Zabiba MSM Al-Dallal HAH Hashim KH : A new technique to solve the maximization of the transportation problems. *AIP Conf. Proc.* 2023;2414:040042. 10.1063/5.0114806

[23] Mohsin M Ahmad M Khan R : Comparative study of metaheuristics in renewable forecasting. *Energies.* 2024;17(9):3158. 10.3390/en17093158

[24] Li Z Chen J Zhang L : Comparison of neural architectures for solar forecasting: Bat-NN vs GRNN. *Renew. Energy.* 2018;127:826–837.

[25] Abbas IT Ghayyib MN : Using sensitivity analysis in linear programming with practical physical applications. *Iraqi J. Sci.* 2024;907–922. 10.24996/ijs.2024.65.2.27

[26] National Aeronautics and Space Administration (NASA): POWER Data Access Viewer. (accessed 2025). Reference Source

[27] National Renewable Energy Laboratory (NREL): PV Watts Calculator. (accessed 2025). Reference Source

[28] Abbas IT Aziz AAH : HMPCS–ML Raw Dataset for Solar Power Forecasting (Version 1.0).[Data set]. *Zenodo.* 2025. 10.5281/zenodo.17560035

[29] Zheng Y Liu Q Chen E : Exploiting temporal dependencies in multivariate time series with LSTM. *IEEE Trans. Neural Netw. Learn. Syst.* 2021;32(9):4022–4032. 10.1109/TNNLS.2020.3015155

[30] Ke G Meng Q Finley T : LightGBM: A highly efficient gradient boosting decision tree. *Adv. Neural Inf. Process. Syst.* 2017;30:3146–3154. Reference Source

[31] Hochreiter S Schmidhuber J : Long short-term memory. *Neural Comput.* 1997;9(8):1735–1780. 10.1162/neco.1997.9.8.1735 9377276

[32] Greff K Srivastava R Koutník J : LSTM: A search space odyssey. *IEEE Trans. Neural Networks Learn. Syst.* 2017;28(10):2222–2232. 27411231 10.1109/TNNLS.2016.2582924

[33] Gers FA Schmidhuber J Cummins F : Learning to forget: Continual prediction with LSTM. *Neural Comput.* 2000;12(10):2451–2471. 11032042 10.1162/089976600300015015

[34] Chen L Zhao X Liu H : Application of LightGBM for renewable energy forecasting with uncertainty quantification. *Energy Rep.* 2023;9:11452–11465.

[35] Little RJ Rubin DB : *Statistical Analysis with Missing Data.* 3rd ed. Wiley;2020.

[36] Iglewicz B Hoaglin DC : *How to Detect and Handle Outliers.* ASQC Quality Press;1993.

[37] Jain AK Dubes RC : Algorithms for data normalization and feature scaling in machine learning. *Pattern Recognit. Lett.* 2023;169:64–72.

[38] Goodfellow I Bengio Y Courville A : *Deep Learning.* MIT Press;2016.

[39] Pascanu R Mikolov T Bengio Y : On the difficulty of training recurrent neural networks. *Proceedings of ICML.* 2013;1310–1318. 10.48550/arXiv.1211.5063 Reference Source

[40] Abbas IT Aziz AAH : Synthetic datasets for HMPCS–ML-based solar power forecasting. *Zenodo.* 2025. 10.5281/zenodo.17432485

