# Supplementary material for: Enhancing Solar Power Forecasting Accuracy Using HMPCS and Machine Learning Techniques: An Applied Study
Source: F1000Res. 2026 Sep 3;15:128. Originally published 2026 Jan 28. [Version 3] doi: 10.12688/f1000research.172121.3 (PMC13589176; doi:10.12688/f1000research.172121.3)
Supplement: Peer review history

# Reviewer report: Reviewer response for version 3

**DOI:** 10.5256/f1000research.208113.r517523

**Published:** 2026-09-18

**Authors:** Ashif Mohammad

**Affiliations:** 1 Bangladesh Betar, Dhaka, Bangladesh

I have reviewed the revised version of the manuscript and the authors’ responses to the previous reviewer comments. The authors have addressed the major concerns by improving the clarity of the methodology, adding explanations regarding the HMPCS framework, expanding the discussion of comparative studies, and providing additional details on experimental evaluation and limitations.

 The revised manuscript is substantially improved in terms of organization and presentation. The authors have also clarified the distinction between standard equations and their proposed methodological contributions, which improves the transparency of the work.

 I have no further major comments at this stage. Minor editorial improvements, such as careful proofreading for grammatical errors, typographical issues, and consistency of terminology throughout the manuscript, may still be considered before final indexing.

Is the work clearly and accurately presented and does it cite the current literature?

Partly

If applicable, is the statistical analysis and its interpretation appropriate?

Partly

Are all the source data underlying the results available to ensure full reproducibility?

Partly

Is the study design appropriate and is the work technically sound?

Partly

Are the conclusions drawn adequately supported by the results?

Partly

Are sufficient details of methods and analysis provided to allow replication by others?

Partly

Reviewer Expertise:

AI, Macine Learning, Solar

I confirm that I have read this submission and believe that I have an appropriate level of expertise to confirm that it is of an acceptable scientific standard.

# Reviewer report: Reviewer response for version 3

**DOI:** 10.5256/f1000research.208113.r517524

**Published:** 2026-09-14

**Authors:** Bin Du

**Affiliations:** 1 Southeast University, Nanjing, Jiangsu, China

The authors have made substantial revisions to the manuscript in response to the reviewers’ comments and have added considerable new content. Each reviewer comment has been addressed point by point, and the responses provided are generally clear and effective in resolving the concerns raised during the previous round of review. The revised manuscript further strengthens and improves the research content on the basis of the original version and provides a more comprehensive and coherent presentation of the work. In particular, the revised manuscript presents the research in a complete, well-organized, and logically structured manner, allowing readers to clearly understand how the study was conducted, what research gap the work aims to address, and what advances have been achieved compared with previous studies. The manuscript is well written and follows appropriate academic conventions. The literature review covers a broad range of relevant studies, while the figures and tables are clear and well integrated with the corresponding textual descriptions, effectively conveying the main ideas and finding of the study. The research objectives and novel aspects of the work are described in sufficient detail, and the research methodology and procedures are presented in a clear and step-by-step manner. The results obtained appear to be reliable and credible. Overall, the study provides useful contribute to both the engineering application and theoretical development of the research field. Based on the substantial revisions and improvements made by the authors, I think that the manuscript is now in a condition suitable for indexing.

Is the work clearly and accurately presented and does it cite the current literature?

Partly

If applicable, is the statistical analysis and its interpretation appropriate?

Partly

Are all the source data underlying the results available to ensure full reproducibility?

Yes

Is the study design appropriate and is the work technically sound?

Yes

Are the conclusions drawn adequately supported by the results?

Yes

Are sufficient details of methods and analysis provided to allow replication by others?

Yes

Reviewer Expertise:

Renewable Energy, Artificial Intelligence, Solar energy, energy systems modeling and optimization, energy storage

I confirm that I have read this submission and believe that I have an appropriate level of expertise to confirm that it is of an acceptable scientific standard.

# Reviewer report: Reviewer response for version 2

**DOI:** 10.5256/f1000research.198686.r505706

**Published:** 2026-08-05

**Authors:** Bin Du

**Affiliations:** 1 Southeast University, Nanjing, Jiangsu, China

The manuscript developed an advanced hybrid model by integrating the hybridization of multi-population cuckoo search (HMPCS) algorithm with machine learning (ML) methods to predict solar power. The superiority of the proposed model was demonstrated through comparisons of various statistical performance indicators. However, I believe that the following suggestions and questions should be addressed before the manuscript can be reconsidered for indexing.

1. the manuscript repeatedly uses the terms “solar power forecasting”, “renewable energy prediction”, “PV forecasting” and “photovoltaic power generation forecasting”. Could the authors clarify the differences among these concepts in the context of the present study. Specifically, what does each term refer to, and what role does each play in the proposed research framework. For the sake of consistency and clarify, the authors are also encouraged to adopt unified terminology through out the manuscript where appropriate;
2. It appears that table 2 is not adequately introduced or discussed in the main text. The authors are encouraged to provide a clear description of the purpose of this table and discuss its key contents and significance to improve the readability and logical flow of the manuscript;
3. The manuscript presents a number of equations and computational procedures in the sections entitled “Data preprocessing equations”, “LSTM model equations”, “HMPCS optimization equations” and “evaluation metrics”. It is not clear whether these equations were newly derived by the authors or adopted from previous studies. If they are original derivations, the authors should provide a brief derivation or theoretical justification. If they are based on existing methods, the corresponding references should be properly cited to acknowledge the original sources;
4. In the introduction, the authors are encouraged to include a brief outline of the manuscript by summarizing the organization of the remaining sections and the main work presented in each section. Such an overview would improve the readability of the manuscript and help readers better understand the overall structure of the study;
5. The reported values of the performance metrics ( *R* *2* ) in several tables range from approximately 0.80 to 0.93. although these results indicate reasonably good predictive performance, they do not appear to be particularly high compared with those reported in some recent studies on photovoltaic (PV) power forecasting, where higher prediction accuracies have been achieved. The authors are encouraged to provide a more detailed comparison with representative studies from the literature and discuss the possible reasons for the differences in predictive performance. Such a discussion would help readers better understand the advantages and limitations of the proposed method.

Please send the revised manuscript to reviewers to review again.

Is the work clearly and accurately presented and does it cite the current literature?

Partly

If applicable, is the statistical analysis and its interpretation appropriate?

Partly

Are all the source data underlying the results available to ensure full reproducibility?

Yes

Is the study design appropriate and is the work technically sound?

Yes

Are the conclusions drawn adequately supported by the results?

Yes

Are sufficient details of methods and analysis provided to allow replication by others?

Yes

Reviewer Expertise:

Renewable Energy, Artificial Intelligence, Solar energy, energy systems modeling and optimization, energy storage

I confirm that I have read this submission and believe that I have an appropriate level of expertise to confirm that it is of an acceptable scientific standard, however I have significant reservations, as outlined above.

# Response

**Published:** 2026-08-05

**Authors:** iraq abbas

**Affiliations:** Mathematics, University of Baghdad Al-Jaderyia Campus College of Science, Baghdad, Baghdad Governorate, Iraq

**We sincerely thank the reviewer for the careful evaluation of our manuscript and for the constructive comments. We have carefully revised the manuscript according to all suggestions. Specifically, we have:**

- Unified the terminology throughout the manuscript and added a terminology clarification section.
- Expanded the discussion of Table 2.
- Clarified the originality of the equations and added the appropriate references.
- Added an "Organization of the Paper" paragraph at the end of the Introduction.
- Included a comparison with recent photovoltaic forecasting studies and expanded the discussion of the obtained R² values.

**All comments have been fully addressed in Version 3 of the manuscript. We sincerely appreciate the reviewer's valuable suggestions, which have significantly improved the quality and clarity of our work.**

# Reviewer report: Reviewer response for version 1

**DOI:** 10.5256/f1000research.189816.r459339

**Published:** 2026-03-02

**Authors:** Ashif Mohammad

**Affiliations:** 1 Bangladesh Betar, Dhaka, Bangladesh

This study proposes a Hybrid Multi-Population Cuckoo Search (HMPCS) framework integrated with machine learning models (LSTM and LightGBM) for improving photovoltaic (PV) power forecasting accuracy. The authors compare HMPCS-optimized models against Grid Search and Particle Swarm Optimization (PSO). Results show that LSTM+HMPCS achieves the lowest RMSE (0.139) and highest R² (0.93), while LightGBM+HMPCS reduces RMSE by 23% relative to unoptimized models (Table 3, p. 9)

 1. Clarity and Use of Literature — **Partly**

 Strengths

- The introduction references recent DL/Transformer-based forecasting studies (2023–2024), indicating awareness of current literature.
- Metaheuristic background is supported with multiple citations.
- A structured literature review is presented (Section 2).

Concerns

- Several grammatical issues and unclear technical phrasing reduce clarity.
- Some citations appear misaligned or weakly integrated into the argument.
- The literature gap is stated but not rigorously justified through systematic comparison.
- Minor typographical inconsistencies (e.g., duplicated or unclear wording in Sections 1–3).

Required Revisions (Mandatory)

- Substantial language editing.
- Stronger synthesis of literature gap (explicit comparison table vs state-of-the-art Transformer-based works).
- Clearer explanation of novelty beyond “multi-population + ML.”

2. Study Design and Technical Soundness — **Partly**

 Strengths

- Logical workflow: preprocessing → model building → optimization → evaluation.
- Explicit HMPCS parameter settings provided (Section 3.8).
- Comparative evaluation against Grid Search and PSO.

Major Concerns

1. **Data Inconsistency**
  - Section 3.5 states meteorological data were sourced from NASA POWER and PV Watts.
  - However, the Data Availability section states the dataset is synthetic and simulated (p. 11)
    ae0a0900-6882-443d-8cb0-c0bde5d…
    .
  - This contradiction must be clarified.
2. **Lack of Cross-Validation Description**
  - The manuscript mentions cross-validation in the fitness function but does not specify:
    - Number of folds
    - Temporal cross-validation strategy (rolling origin?)
    - Random seed control
3. **No Statistical Significance Testing**
  - Performance improvements are reported descriptively.
  - No hypothesis testing, confidence intervals, or repeated runs analysis.

Required Revisions (Mandatory)

- Clarify whether data are real, synthetic, or hybrid.
- Specify validation protocol clearly.
- Provide statistical robustness analysis (e.g., multiple runs, standard deviations).

3. Replicability — **Partly**

 Strengths

- Zenodo repository links provided.
- Hyperparameter settings listed.
- Evaluation metrics clearly defined.

Missing Critical Details

- Dataset size (number of samples) not clearly specified.
- Training epochs and batch sizes not reported.
- Hardware specifications missing.
- No ablation study to isolate HMPCS contribution.
- Convergence curves are mentioned but insufficiently documented.

Required Revisions (Mandatory)

- Provide full experimental configuration table.
- Add reproducibility checklist.
- Include ablation analysis (e.g., single-population vs multi-population).

4. Statistical Analysis — **Partly**

 Strengths

- Standard regression metrics (RMSE, MAE, MAPE, R²) used appropriately.
- Comparative table (Table 3) is clear.

Weaknesses

- No uncertainty quantification.
- No repeated trials reported.
- No statistical test comparing optimizers.
- Percentage improvement claims lack statistical backing.

Required Revisions (Mandatory)

- Perform ≥10 independent runs per optimizer.
- Report mean ± standard deviation.
- Conduct statistical significance test (e.g., Wilcoxon signed-rank test).

5. Data Availability — **Partly**

 Zenodo links are provided (p. 11)

 ae0a0900-6882-443d-8cb0-c0bde5d…

 , which is positive.

 However:

- Data described as “synthetic and simulated.”
- Yet earlier sections imply NASA/PV Watts sourcing.
- Reproducibility depends on clarity regarding data generation process.

Required Revision

- Provide full data generation protocol.
- Clarify real vs synthetic usage.
- Include dataset dimensions and time span.

6. Conclusions — **Partly**

 The conclusions align directionally with reported results.

 However:

- Claims regarding robustness and real-world grid deployment are speculative.
- No computational time comparison table provided.
- Policy implications are overstated relative to experimental scope.

Required Revisions

- Temper policy and deployment claims.
- Add computational efficiency comparison.
- Explicitly state study limitations.

Minor Issues

- Typographical inconsistencies.
- Occasional unclear phrasing in equations formatting.
- Some redundant explanation of evaluation metrics.
- Figures lack detailed captions and axis labeling explanation.

Overall Recommendation

 Major Revision Required

 The study has **promising technical contribution**, but before being scientifically sound, it must:

1. Resolve data inconsistency (real vs synthetic).
2. Strengthen reproducibility and statistical robustness.
3. Provide proper validation protocol.
4. Include statistical testing.
5. Clarify novelty relative to recent Transformer-based PV forecasting literature.
6. Improve language and technical precision.

Is the work clearly and accurately presented and does it cite the current literature?

Partly

If applicable, is the statistical analysis and its interpretation appropriate?

Partly

Are all the source data underlying the results available to ensure full reproducibility?

Partly

Is the study design appropriate and is the work technically sound?

Partly

Are the conclusions drawn adequately supported by the results?

Partly

Are sufficient details of methods and analysis provided to allow replication by others?

Partly

Reviewer Expertise:

EnergySolar EnergyArtificial IntelligenceMPPT

I confirm that I have read this submission and believe that I have an appropriate level of expertise to state that I do not consider it to be of an acceptable scientific standard, for reasons outlined above.

# Response

**Published:** 2026-04-10

**Authors:** iraq abbas

**Affiliations:** Mathematics, University of Baghdad Al-Jaderyia Campus College of Science, Baghdad, Baghdad Governorate, Iraq

We sincerely thank the reviewer for the valuable and constructive feedback.

 All comments have been carefully addressed in the revised version of the manuscript. In particular, we have clarified the data sources by distinguishing between real-world datasets (NASA POWER and PVWatts) and synthetic data used for robustness analysis. We have also strengthened the methodological section by clearly defining the validation protocol, including time-series cross-validation and repeated experimental runs.

 Furthermore, statistical robustness has been improved by reporting mean ± standard deviation over multiple runs and by applying the Wilcoxon signed-rank test to confirm the significance of the observed improvements.

 Additional enhancements include a detailed experimental setup, an ablation study to isolate the contribution of the HMPCS framework, and improvements in language, clarity, and presentation. The conclusions have also been revised to better align with the experimental scope, including a clear discussion of limitations and computational efficiency.

 We believe that these revisions have significantly improved the quality, clarity, and reproducibility of the manuscript.
